# Molecular mimicry in the pathogenesis of autoimmune rheumatic diseases

**DOI:** 10.1016/j.jtauto.2025.100269

**Published:** 2025-01-07

**Authors:** Michaela Fehringer, Thomas Vogl

**Affiliations:** Medical University of Vienna, Borschkegasse 8a, 1090, Vienna, Austria

**Keywords:** Rheumatoid arthritis, Systemic lupus erythematosus, Ankylosing spondylitis, Systemic sclerosis, Myositis, Molecular mimicry

## Abstract

Autoimmune rheumatic diseases (ARDs) are a heterogeneous group of conditions characterized by excessive and misdirected immune responses against the body's own musculoskeletal tissues. Their exact aetiology remains unclear, with genetic, demographic, behavioural and environmental factors implicated in disease onset. One prominent hypothesis for the initial breach of immune tolerance (leading to autoimmunity) is molecular mimicry, which describes structural or sequence similarities between human and microbial proteins (mimotopes). This similarity can lead to cross-reactive antibodies and T-cell receptors, resulting in an immune response against autoantigens. Both commensal microbes in the human microbiome and pathogens can trigger molecular mimicry, thereby potentially contributing to the onset of ARDs.

In this review, we focus on the role of molecular mimicry in the onset of rheumatoid arthritis and systemic lupus erythematosus. Moreover, implications of molecular mimicry are also briefly discussed for ankylosing spondylitis, systemic sclerosis and myositis.

## 1 Introduction

Autoimmune rheumatic diseases (ARDs) represent a heterogeneous group of conditions characterized by exaggerated self-reactive, antigen-driven immune responses. These aberrant responses typically target tissues within the musculoskeletal system, including joints, bones, muscles, entheses, as well as internal organs [1,2].

ARDs affect an estimated 5 % of the general population [3], with a notable increase in prevalence observed in recent decades [4]. The aetiology of ARDs is complex and multifactorial [5], involving genetic, demographic, behavioural and environmental factors. Genetic predisposition is evidenced by the high prevalence of ARDs among family members and the identification of high-risk loci in genetic studies [[6], [7], [8]]. Meanwhile, demographic factors include female sex, age and lifestyle, with women having a two to nine times higher risk of developing ARDs, depending on the disease [9]. Moreover, it was observed that autoimmune diseases tend to occur in the second half of adulthood due to immune ageing and reduced immune competence [10,11]. Developed countries, characterized by increased hygiene and westernized diet, also have higher rates of autoimmune diseases [[12], [13], [14]]. In addition, behavioural factors, such as smoking, further contribute to disease risk [15].

Environmental factors, particularly microbial colonization and infections, are in the focus of this review, as they might contribute to the observed rise in ARD prevalence [1,[16], [17], [18]]. Microbes can induce autoimmunity through a variety of mechanisms, with molecular mimicry being the most prominent [17,19].

### 1.1 Antigen recognition by and cross-reactivity of adaptive immune receptors

The human adaptive immune system detects a vast array of antigens either via T-cell receptors (TCRs) or immunoglobulins (Igs), encoded by B-cell receptors (BCRs). Intracellular or extracellular antigens are presented for detection by the TCR on the surfaces of the body's own cells via major histocompatibility complex (MHC) class II for CD4^+^ T-cells, or MHC class I for CD8^+^ T-cells, respectively [20]. These peptides can originate from larger proteins that have been digested or processed, rendering the amino acid sequence more critical than the structure [21,22]. In contrast, Igs, also known as antibodies, primarily recognize epitopes exposed on the surface of unprocessed molecules. Consequently, antibody binding depends not only on amino acid sequence but also on structural similarity [22].

The adaptive immune system generates antibody and TCR diversity through the process of somatic VDJ recombination, where antigen receptor segments are randomly cleaved and re-joined [23,24]. Nevertheless, the immune system needs to recognize >10^15^ potential foreign peptides [25]. If each T-cell recognized only one single peptide, the required number of T-cells would weigh more than 500 kg [26], which is clearly unrealistic. Cross-reactivity (where one receptor recognizes multiple antigens) has potentially evolved as a solution: There are <10^8^ distinct TCRs estimated in the naïve T-cell pool [27], and each single TCR is estimated to recognize up to 10^6^ different peptides [22,25,28]. Additionally, there are about 10^11^ different BCRs on naïve B-cells [29,30], which undergo somatic hypermutation to increase diversity, however, cross-reactivity is nevertheless necessary to detect all foreign peptides [31].

Thus, cross-reactivity of the adaptive immune receptors to sequence- or structurally related epitopes has evolved as a strategy to recognize (nearly) all pathogenic antigens, enabling the initiation of immune responses aimed at their elimination. Rather than a malfunction, cross-reactivity represents a nuanced trade-off, reflecting the versatility of the adaptive immune system in protecting from a broad range of pathogens [22,[32], [33], [34]].

### 1.2 The human microbiome

The human microbiome, consisting of the diverse microbial communities living in and on the body, includes approximately 10–100 trillion microbial cells per person [35]. While most of these microbes (microbiota) reside in the gut, they also inhabit the skin, mouth, respiratory and urogenital tract, collectively equalling the number of human cells in the body [36]. These microbes perform essential functions such as extracting otherwise inaccessible nutrients and energy from the diet, as well as synthesizing vitamins [[37], [38], [39], [40], [41]]. They are also recognized by the immune system, contributing to its maturation and activity [42,43], and may potentially trigger disease-causing mechanisms such as molecular mimicry.

### 1.3 Molecular mimicry in autoimmune rheumatic diseases

**Fig. 1:**
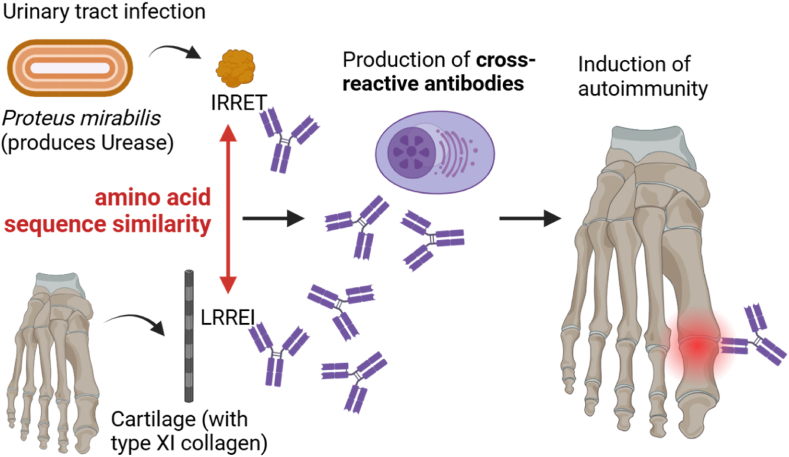
**Principle of molecular mimicry, exemplified on*****Proteus mirabilis*****colonization.** Pathobionts and pathogens can produce antigens with similarity to self-antigens (mimotopes), be it through sequence or structural similarity. This can trigger molecular mimicry and thus induce the production of cross-reactive T-cells or antibodies that bind to both structures, thereby inducing autoimmunity. Autoimmunity can persist even after the microbe has been eliminated by the immune system. Created in BioRender.com.

Molecular mimicry stands as a prominent theory explaining the initiation of autoimmune diseases in genetically predisposed individuals. It describes the structural or sequence similarity between exogenous peptides and self-peptides, and the exogenous peptides mimicking self-peptides are termed ‘mimotopes’ [17,18,22,33,44,45]. These mimotopes can stimulate cross-reactive T- or B-cells, leading to an initial breakdown in self-tolerance and triggering an immune response against self-antigens (Fig. 1). Mimotopes can originate from pathobionts – symbiotic microbiota that may turn pathogenic under certain conditions [46,47] – or pathogens and may be part of the microbe's structure or secreted by it.

From an evolutionarily perspective, microbes may use molecular mimicry to disguise themselves as self-antigens, potentially evading clearance mechanisms and promoting their survival [22,33,34,44]. However, if molecular mimicry leads to an excessive autoimmune reaction and systemic inflammation as observed in autoimmune diseases, it may trigger pro-inflammatory cascades and antimicrobial responses, potentially overriding tolerance mechanisms and, in extreme cases, causing the host's death [32]. This balance highlights the complex interactions between pathogen survival strategies and host immunity.

Over the last decades, increasing evidence has been linking molecular mimicry to the onset of autoimmune diseases, including multiple ARDs [17,33,34,45,48,49]. This review summarizes the literature on molecular mimicry available for rheumatoid arthritis (RA) and systemic lupus erythematosus (SLE). Moreover, we will briefly touch upon molecular mimicry implications in ankylosing spondylitis (AS), systemic sclerosis (SSc) and myositis. Other mechanisms of pathogenicity through which microbes may induce autoimmunity include microbial translocation, where microbes move from their usual sites to other organs, inducing inflammation, or epitope spreading, where an immune response initially targets a specific antigen but later expands to recognize other epitopes. These and other mechanisms are shortly mentioned at the end of this manuscript (see 5.3 Other mechanisms of pathogenicity).

A significant driver of the increasing knowledge about molecular mimicry has been the integration of bioinformatics and *in silico* approaches into medical research. Early studies on molecular mimicry were limited to specific bacterial or human structures of interest [[50], [51], [52]], providing only a narrow view of microbial involvement. Nowadays, bioinformatic tools enable high-throughput, efficient, and cost-effective methods for identifying potential microbial mimotopes triggering molecular mimicry on a larger scale [[53], [54], [55], [56], [57], [58]].

For example, early on, tools such as the Basic Local Alignment Search Tool algorithm (BLAST; https://blast.ncbi.nlm.nih.gov/Blast.cgi) [59] allowed to compare protein or peptide sequences (e.g. linear T-cell or B-cell epitopes) with large databases, such as those provided by the National Center for Biotechnology Information (NCBI; https://www.ncbi.nlm.nih.gov/) [60] or UniProtKB (https://www.uniprot.org/help/uniprotkb) [61]. This process helps identify sequences with significant similarity. Epitope prediction algorithms, such as those in the Next-Generation Immune Epitope Database (IEDB) Tools website (https://nextgen-tools.iedb.org/) can be used for T-cell and B-cell epitope prediction to identify immunologically relevant mimotopes [62]. Moreover, the binding of epitopes to HLA molecules can be predicted using NetMHC (https://services.healthtech.dtu.dk/services/NetMHC-4.0/) [63,64]. Structural mimotopes can be identified using structural modelling tools, such as SWISS-MODEL (https://swissmodel.expasy.org/) [65] or AlphaFold (https://alphafold.ebi.ac.uk/) [66], which predict three-dimensional protein structures. However, predictions from in silico analyses require validation through experimental approaches, such as immunoassays, functional assays or structural biology techniques, to confirm their relevance.

It is important to note that the associations discussed in this review remain correlations, as causality has not yet been established in humans. For example, patients with ARDs may exhibit higher infection rates with specific microbes due to immunosuppressive therapy [67], rather than the microbe itself triggering autoimmunity. Furthermore, some of the cited studies are older and may not align with current research standards. The authors have aimed to highlight both the limitations of certain studies and the strengths of others throughout the text.

Some of the studies were conducted in mice and rabbits, which serve as valuable models for finding initial interesting associations. However, it is important to recognize that the germline genes of these species partially differ from those in humans, limiting the direct translation of findings from animal models to human autoimmunity [68,69]. Therefore, confirmation in human studies is essential to substantiate these associations.

## 2 Molecular mimicry in rheumatoid arthritis

**Fig. 2:**
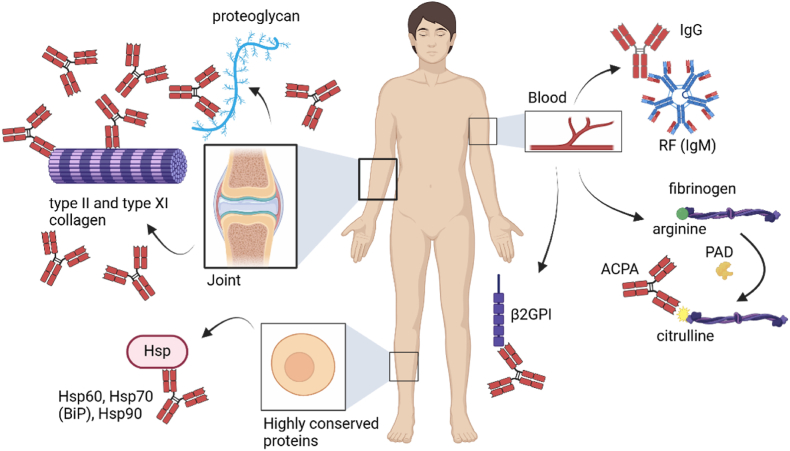
**Autoantibodies inrheumatoidarthritis (RA).** RA is characterized by the presence of rheumatoid factor (RF) and anti-citrullinated protein antibodies (ACPAs), e.g. against citrullinated fibrinogen, in the bloodstream, both of which are used for diagnosis. RF is an IgM antibody that targets the Fc region of IgG, while ACPAs are IgG antibodies that recognize citrullinated epitopes. Citrullination, mediated by the enzyme protein arginine deiminase (PAD), converts arginine into citrulline, a process that is dysregulated in RA. Additionally, anti-β2-glycoprotein I (β2GPI) antibodies are often detected in the blood of RA patients. RA also leads to antibody accumulation in the joints, targeting structures like proteoglycans and type II and type XI collagen. Furthermore, antibodies in RA target conserved proteins such as various heat shock proteins (Hsp). BiP=Binding immunoglobulin protein. Created in BioRender.com.

RA is a chronic, inflammatory joint disease that can lead to synovitis and erosion of articular cartilage and underlying bone, thus inducing joint damage and reduced mobility [16,70]. Additionally, RA can induce systemic symptoms, including an increased risk of lung disease, cardiovascular disease, disorders affecting the peripheral or central nervous systems, and a higher susceptibility to infections [71]. RA affects approximately 0.5–1 % of the world's population [1,16,72] and is characterized by the presence of antibodies targeting autoantigens (Fig. 2), such as rheumatoid factor (RF; targeting the constant region of IgG) [73] or anti-citrullinated protein antibodies (ACPAs; targeting citrulline-containing epitopes on post-translationally modified proteins) [16,[74], [75], [76]]. Citrullination of proteins is mediated by peptidyl arginine-deiminase (PAD) enzymes that transform the amino acid arginine to citrulline, a process that is dysregulated in RA patients [77]. Citrullinated fibrinogen is a typical autoantigen, the autoantibodies against which are highly specific for RA [78,79]. Other autoantigens include proteins associated with the joint [80], such as proteoglycans, type II and type XI collagen [81], or highly conserved proteins such as heat shock proteins (Hsps) [82], with one example being the chaperone binding immunoglobulin protein (BiP) located in the endoplasmic reticulum [83]. Moreover, anti-β2-glycoprotein I (β2GPI) antibodies associated with antiphospholipid syndrome (APS) are common in RA patients [84].

The aetiology of RA is complex, involving both genetic and environmental factors. The most significant genetic association is the presence of specific HLA (=MHC) alleles. While HLA class I is present on all nucleated cells, HLA class II is expressed by antigen-presenting cells (APCs) and presents external peptides to the TCR on CD4^+^ T-cells [85]. The structure of the HLA class II binding pocket influences the repertoire of peptides that can be presented to CD4^+^ T-cells, with certain alleles leading to structural variations that may contribute to disease [85]. In RA, the presence of certain HLA class II alleles, such as HLA-DRB1∗01 (DR1) or HLA-DRB1∗04 (DR4) [18,72,86,87] is linked to disease susceptibility. These high-risk alleles share a common amino acid motif, known as the shared epitope (SE). The SE consists of the amino acid motif QKRAA, QRRAA or RRRAA at position 70–74 according to Wysocki *et al.* [87] or EQRRAA at position 69–74 according to Rashid and Ebringer [18]. Approximately 70 % of RA patients carry HLA-DRB1 alleles containing the SE [86], and it is positively correlated with ACPA-positive RA [88].

The mechanism by which the SE contributes to the production of ACPAs remains unclear [89]. As these residues are located in an α-helix of the antigen-binding cleft [90], a key site for T-cell recognition, it is hypothesized that the SE may affect interactions with CD4^+^ T cells. One theory suggests that SE-positive HLA-DR molecules preferentially bind citrullinated peptides [91].

Another hypothesis, known as the ‘hapten-carrier model’, proposes that B-cells bind to the PAD4/protein complex during citrullination [89,92]. In this model, PAD4 acts as the carrier, while the protein – considered a hapten – is a small molecule that only triggers an immune response when attached to a larger carrier (PAD4). After binding, B-cells process the complex and present a PAD4 peptide to PAD4-specific T-cells. With help from these T-cells, the B-cells differentiate into plasma cells that produce ACPAs. This theory is supported by the observation that RA patients generate T-cells against PAD4 [92].

**Fig. 3:**
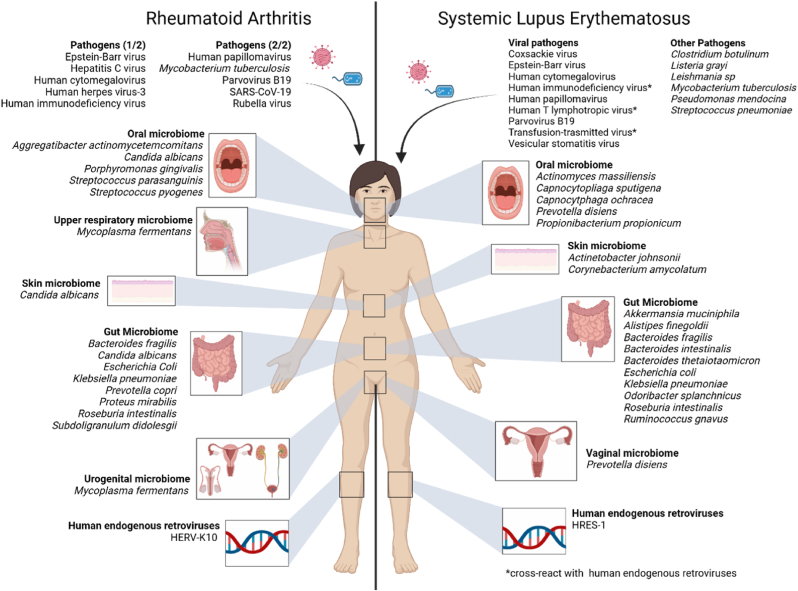
**Summary of all microbes implicated in the pathogenesis of rheumatoid arthritis (RA) and systemic lupus erythematosus (SLE) through molecular mimicry.** Members of the oral, skin, respiratory, gut and urogenital (including vaginal) microbiome have been associated with RA and SLE. Moreover, pathogens and human endogenous retroviruses are implicated. HERV = human endogenous retrovirus, HRES=HTLV (human T-cell lymphotropic virus)-related endogenous sequence. Created in BioRender.com.

Besides this genetic component, microbial colonization/infections have been proposed as one environmental trigger that has the potential to trigger the onset of RA. Specifically, microbes can trigger autoimmune diseases through molecular mimicry. The following sections describe several members of the human microbiome, as well as pathogens where molecular mimicry has been suggested. Specific mimotopes, autoantigens and, if known, their amino acid sequences, associated with RA are listed in Table 1. Moreover, Fig. 3 gives an overview of the discussed mimotopes.

**Table 1:** **Instances of molecular mimicry between microbial mimotopes and autoantigens in rheumatoid arthritis (RA).** This table highlights examples discussed in this review where molecular mimicry has been implicated in RA pathogenesis. Only microbes are included where both the microbial structure (mimotope) and human autoantigen have been identified, and where some form of cross-reactivity has been observed: ‘Level of evidence’ indicates which cross-reactive component of the adaptive immune system recognizes the mimotope or microbe. While not exhaustive, this list provides a representative overview of the most well-established connections between microbial infections and autoimmune responses in RA.

Microbe	Microbial structure	Human structure	Level of evidence	References
*Bacteroides fragilis*	ubiquitin (BfUbb) (KQMEDGRT)	Ubiquitin (KQLEDGRTL)	Antibodies in rabbits	[93]
	ubiquitin (BfUbb) (QDKEGFPPDKIRL)	Ubiquitin (QDKEGIPPDQQRL)	Antibodies in rabbits	[93]
	Hsp70 (3 different peptide mimotopes)	BiP (three different T-cell epitopes)	T-cells (predicted)	[55]
Epstein-Barr-virus	EBNA-1 (EBNA35-58Cit)	Citrullinated fibrin AhFibA peptide (β60-74Cit)	Antibodies in RA patients	[94]
	EBNA-1 (p107)	Denatured collagen and keratin	Antibodies in RA patients	[95]
	EBNA-1 (G/A repeat sequence (P62)	Keratin, actin, type II collagen, cytokeratin, proteoglycans	Antibodies in RA patients	[51,96]
	gp110 (QKRAA)	Shared Epitope (QKRAA) of HLA-DR4	T-cells and antibodies	[50]
	EBNA-6 (Six amino acid sequence)	HLA-DQ∗0302 (6 amino acid sequence)	Not specified	[48]
	vIL-10	IL-10	B-cells	[97,98,99]
*Escherichia coli*	Heat shock protein DnaJ (QKRAA)	SE motif (QKRAA) on HLA-DR alleles	T-cells in RA patients	[100]
	L-asparaginase (IANVKGEQVVN at amino acid position 68–78)	Type II collagen (IAGFKGEQGPK at amino acid position 260–270)	T-cells in RA patients	[101]
Human endogenous retroviral element K10	Gag (15 amino acid sequence including GKELK)	IgG1Fc (GKEYK)	IgG in RA patients	[58]
*Klebsiella pneumoniae*	L-asparaginase (IANVKGEQVVN at amino acid position 68–78)	Type II collagen (IAGFKGEQGPK at amino acid position 260–270)	T-cells in RA patients	[101]
*Mycobacterium* genus	L-asparaginase (IANVKGEQVVN at amino acid position 68–78)	Type II collagen (IAGFKGEQGPK at amino acid position 260–270)	T-cells in RA patients	[101]
*Porphyromonas gingivalis*	ENO1	Enolase	Antibodies in mice and RA patients	[102,103]
*Prevotella copri*	*Prevotella* arylsulfatase protein	N-acetylglucosamine-6-sulfatase (GNS)	Antibodies and T-cells in RA patients	[104]
	Extracellular/secreted Prevotella protein (WP_028897633)	Filamin A (FLNA)	Antibodies and T-cells in RA patients	[104]
	Molecular chaperone DnaK	BiP	T-cell (predicted)	[55]
*Proteus*	L-asparaginase (IANVKGEQVVN at amino acid position 68–78)	Type II collagen (IAGFKGEQGPK at amino acid position 260–270)	T-cells in RA patients	[101]
*Proteus mirabilis*	Haemolysin (Hpm B) (ESSRAL)	SE motif (EQRRAA) of HLA-DR alleles	Antibodies in RA patients	[105,106]
	Haemolysin (SQIRLDLSSPKLT at amino acid position 349–361)	Type II collagen (VEIRAEGNSRFTY at position 1350–1362)	Predicted: T-cells in RA patients	[107]
	Urease (UreC) (IRRET)	Type XI collagen (LRREI)	Antibodies in RA patients	[106]
*Streptococcus* genus	L-asparaginase (IANVKGEQVVN at amino acid position 68–78)	Type II collagen (IAGFKGEQGPK at amino acid position 260–270)	T-cells in RA patients	[101]

### 2.1 Gut microbiome

One gut bacterium with extensive data on molecular mimicry is *Prevotella copri* [108]. This microbe is strongly present in the gut microbiome of new-onset RA patients and individuals in pre-clinical stages of RA [109,110]. Its increased abundance is one of the most significant changes in the gut microbiome of pre-clinical RA patients [45]. *P. copri* can induce arthritis in mice via mono-colonization [111,112], and RA patients have increased antibody responses against at least five *P. copri* peptides [[113], [114], [115]]. *P. copri* produces mimotopes of self-epitopes, especially in patients with SE alleles [104]. Specifically, two HLA-DR-presented autoantigens, N-acetylglucosamine-6-sulfatase (GNS) and filamin A (FLNA), have been identified as targets in RA patients, with high sequence similarity found in *P. copri* proteins [104]. Additionally, *P. copri* increases the immune response to the autoantigen RLP23A, a human ribosomal protein, possibly through molecular mimicry [112]. In a recent bioinformatic analysis, *Prevotella* sp., including *P. copri*, were predicted to produce mimotopes to a T-cell epitope of BiP [55]. In *P. copri* specifically, the protein containing the predicted mimotope is the molecular chaperone DnaK. As for all bioinformatically predicted mimotopes, validation through cross-reactivity studies involving lymphocytes or antibodies is essential.

Another bacterium with substantial evidence linking it to RA aetiology is *Proteus mirabilis* [116]. *P. mirabilis*, part of the gut microbiome, is known to cause urinary tract infections [117]. It is the most prominent microbe found in the urine of RA patients, who also show increased antibody levels against it [105]. Chronic infection by *P. mirabilis* can induce chronic inflammation of joints, presumably through molecular mimicry [34,118]: The two bacterial peptides hemolysin B (Hpm B) and Urease C (UreC) share sequence similarity to the SE and type XI collagen [105,106], respectively. Ebringer *et al.* [105] hypothesized that the sequence similarity to Hpm B could induce antibodies against HLA-DR alleles containing the SE, though this has not been elucidated yet. Moreover, Ebringer *et al.* [119] note that the mimotopes as well as the autoantigens contain target sites for PADs, suggesting this could play a role in the generation of polyclonal ACPAs. Another study showed that RA patients have increased antibodies against Hpm B and UreC [118]. Hemolysin was additionally shown to have a similar sequence to type II collagen [107]. Moreover, it was bioinformatically predicted that *P. mirabilis* produces a mimotope of a T-cell epitope of the HLA class I histocompatibility antigen B α chain [55]. The *Proteus* genus was also shown to produce bacterial L-asparaginase that shares an amino acid sequence with an immunodominant T-cell epitope on type II collagen [101]. This bacterial mimotope can activate DR4-restricted T-cells and induce the production of pro-inflammatory cytokines [101].

*Bacteroides fragilis*, also an opportunistic pathogen, produces a protein named *B. fragilis* ubiquitin (BfUbb) that shows high structural and sequence similarity to human ubiquitin and is recognized by antibodies targeting human ubiquitin [93,120]. RA patients were shown to exhibit higher antibody responses against BfUbb than healthy controls. In a metagenomic shotgun sequencing and metagenome-wide association study, Zhang *et al.* [121] showed that several suggested mimotopes of type XI collagen and HLA-DR4/1 produced by the *Bacteroides* genus are increased in RA gut samples. It was further predicted through bioinformatic analysis that *B. fragilis* produces an Hsp70 protein that contains three mimotopes to three different T-cell epitopes of BiP [55].

*Escherichia coli* expresses the Hsp DnaJ containing a similar sequence to the SE [100]. T-cells from RA patients with SE alleles recognize and proliferate in response to DnaJ [100]. Similar to *Proteus* sp., also *E. coli* expresses the protein L-asparaginase with sequence similarity to a T-cell epitope of type II collagen [101]. Moreover, it is suggested to express several mimotopes of the HLA-DR4/1 that were found to be enriched in RA gut samples [121]. Interestingly, it was predicted through bioinformatic analysis that *E. coli* produces ∼100 potential mimotopes of T-cell epitopes, specifically of type II collagen, the DR4 beta chain, HLA-B (of the MHC class I), fibrinogen and BiP [55]. In another bioinformatic analysis, *E. coli* was predicted to produce the same sequence triggering molecular mimicry of type XI collagen that *P. mirabilis* produces [106].

Chriswell *et al.* [74] recently identified *Subdoligranulum didolesgii*of the *Ruminococcaceae* family in an insightful study, demonstrating that the bacterium is recognized by IgA/IgG antibodies and activates CD4^+^ T-cells in RA patients. Moreover, they discovered that *S. didolesgii* can cause autoantibody production and joint disease in a mouse model. Cross-reactivity between *S. didolesgii* and RA-related autoantigens suggests molecular mimicry as a possible pathogenic mechanism. This is further supported by Zhang *et al.* [121] who found that a protein of the *Subdoligranulum* genus that is suggested to mimic HLA-DR4/1 is enriched in the gut of RA patients.

Other instances of molecular mimicry in gut microbes include the following: Cross-reactivity between *Roseburia intestinalis* DNA cytosine methyltransferase and β2GPI results in anti-β2GPI autoantibody production [122]. RA patients have a higher frequency of IgA anti-β2GPI antibodies, potentially induced by cross-reaction with *R. intestinalis*, contributing to RA pathogenesis [45]. Moreover, Zhang *et al.* [121] could show that a mimotope of type XI collagen produced by the *Roseburia* genus in enriched in the gut of RA patients.

Additionally, the gut microbe *Klebsiella pneumoniae* (that can also be found in the oropharynx and turn pathogenic) produces the protein L-asparaginase triggering molecular mimicry of type II collagen [101], as well as a mimotope of HLA-DR4/1 enriched in RA patients [121]. It was predicted through bioinformatic analysis that *K. pneumoniae* produces several mimotopes of T-cell epitopes, specifically of collagen α-1(II) chain, HLA class I histocompatibility antigen B α chain, keratin type I cytoskeletal 18, fibrinogen beta chain, BiP, and α-enolase (ENO1) [55].

While as of now only bacteria have been discussed, also a fungus may be mentioned: *Candida albicans*, that can be found in the intestines as well as the mouth and skin and that may turn pathogenic [123], is also predicted to produce mimotopes of several T-cell epitopes of BiP [55].

### 2.2 Oral microbiome

*Porphyromonas gingivalis* is a major pathogenic bacterium in periodontal diseases that is correlated with RA onset [90,[124], [125], [126], [127], [128]]. Mono-colonization with this bacterium induces arthritis in a mouse model [129]. It is correlated with increased RA-specific antibody titres against the autoantigen human ENO1 [130], possibly due to molecular mimicry between the bacterial *P. gingivalis* enolase and ENO1, which share 82 % sequence similarity at the immunodominant regions [131,102]. Moreover, mimotopes of type XI collagen and HLA-DR4/1 produced by the *Porphyromonas* genus have been identified to be enriched in the gut of RA patients [121]. *P. gingivalis* is, to date, the only recorded microbe able to citrullinate bacterial or human proteins through its bacterial PAD, thereby triggering the production of ACPAs that cross-react with human citrullinated peptides through molecular mimicry [101,128].

A recent study has shown that several oral microbes including *P. gingivalis* may become citrullinated in the oral cavity through the action of bacterial or human PADs [132]. The citrullinated microbial proteins are then bound by B-cells, which subsequently undergo affinity maturation and epitope spreading to bind citrullinated human antigens. This process provides a plausible explanation for the generation of ACPAs through molecular mimicry between citrullinated microbial and self-peptides. Other bacteria in which this phenomenon was observed include *Streptococcus parasanguinis* or *Aggregatibacter actinomycetemcomitans* [132].

The *Streptococcus* genus is generally implicated in RA, as it can trigger a specific type of arthritis called post-streptococcal reactive arthritis (PSRA) [133]. *Streptococcus* produces L-asparaginase, which mimics type II collagen [101], and assumed mimotopes of type XI collagen autoantigen and HLA-DR4/1 that are enriched in RA patients [121]. Another species, *Streptococcus pyogenes*, was predicted to produce several mimotopes of a T-cell epitope of type II collagen [55]. *A. actinomycetemcomitans* is strongly implicated in RA aetiology due to its role in driving dysregulated citrullination of self-proteins and the generation of citrullinated autoantigens targeted in RA [134]. Moreover, the *Aggregatibacter* genus was found to produce proteins suggested to be mimotopes of type XI collagen and HLA-DR4/1 that are enriched in RA patients [121].

### 2.3 Urogenital and respiratory microbiome

It has been observed that the diversity of the lung microbiota decreases in RA patients [135]. However, the role of molecular mimicry in the genital and lung microbiome during RA aetiology remains relatively unexplored. One noteworthy microbe studied in this context is *Mycoplasma fermentans* [136], a bacterium that has been found in the urogenital and upper respiratory tract and is potentially pathogenic. Major *M. fermentans* antigens were found in RA patients’ tissues [136], and *Mycoplasma* species are indicated to trigger molecular mimicry [[137], [138], [139]], however, additional research is needed to further elucidate the role of these microbes.

### 2.4 Pathogens

Epstein-Barr virus (EBV) is an infectious agent causing infectious mononucleosis that has infected more than 98 % of the world's population by the age of 40 [48,72]. EBV is associated with several autoimmune diseases (see 3.3 Pathogens, 4.2 Systemic Sclerosis and 5.1 The role of different microbe populations). Studies show that EBV is found in increased titres in the synovial tissues of RA patients [140] and that anti-EBV titres are ten-fold higher in patients with RA compared to healthy controls [141]. Furthermore, RA patients exhibit serum reactivity against EBV nuclear antigen-1 (EBNA-1) [142]. EBNA-1 contains a repeated glycine-alanine sequence, which it shares with the SE. Various epitopes of EBNA-1 are suggested to mimic several autoantigens in RA, such as cytokeratin, type II collagen, actin, keratin and citrullinated fibrin [33,51,72,96,94]. Moreover, the viral glycoprotein gp110 shares an epitope with sequence similarity to the SE [50], with B- and T-cells shown to cross-react with both epitopes [48]. The EBNA-6 protein contains a mimotope of HLA-DQ∗0302 [48], a HLA class II molecule associated with increased autoimmunity susceptibility [143], and mimotopes of IL-10, CXCR and IL-12p40 have also been described [[97], [98], [99], [144]]. Finally, EBV shows sequence similarity to the peptide TLRVYK that is recognized by anti-β2GPI antibodies [145].

Parvovirus B19 (PB19) is another important pathogen, as infection may trigger rheumatic disease phenotype, potentially even permanently [146]. Its DNA can be detected in the synovial fluid and cells of affected joints of RA patients [147], and it triggers autoantibody production against a vast array of autoantigens including nuclear antigens, RF and others. The existence of cross-reactive anti-viral antibodies has been established: Anti-viral IgG antibodies from patients with persistent PB19 infection cross-react with several autoantigens, including human keratin and type II collagen [148]. Moreover, mice developed antibodies against these autoantigens when immunized with a PB19 peptide [148].

Another potential trigger is severe acute respiratory syndrome coronavirus type 2 (SARS-CoV-2), the virus causing COVID-19, since an increased onset of RA is observed after infection [149]. The reason could be molecular mimicry, as sequence identity between several viral and human peptides (39 % between SARS-CoV-2 ORF1ab and the potential autoantigen poly ADP-ribose polymerase 14, and 20 % with the protein mono-ADP-ribosyltransferase PARP9 isoform D) has been reported. Another study predicted bioinformatically that ORF1ab encompasses 18 matching peptides identical to peptides of the human proteome, along with some other peptides showing sequence similarity [56].

Also, the *Mycobacterium* genus is implicated as it was shown to produce L-asparaginase, as already described [101]. Mycobacterial Hsp65 shows sequence similarity to human Hsp60, and elevated T-cell and antibody responses to the mimotope have been demonstrated in RA patients [150,151]. However, the study on elevated T-cell responses was only performed on a single patient [150]. Another study could show that T-cells from RA patients reactive to human Hsp60 also recognize mycobacterial Hsp65 [152]. Again, this study was only performed on two patients and both studies thus require re-validation with a bigger patient cohort. Moreover, *Mycobacterium tuberculosis* produces a mimotope that is recognized by cross-reactive T-cells from RA patients and rats suffering from adjuvant arthritis [52]. The peptide triggering molecular mimicry is cross-reactive with a self-antigen in joint cartilage.

Rubella virus (that has been detected in synovial joints preceding arthritis episodes [153]) and human herpes virus (HHV)-3 (that is increased in RA patients [154]) were shown to produce a structural polyprotein and a gene 20 protein, respectively, that contain predicted mimotopes of type II collagen [107]. Human papillomavirus (HPV), together with other microbial agents, has been predicted to produce the amino acid motif IRRET (as *P. mirabilis* and its UreC) that shows sequence similarity to type XI collagen [155]. Human cytomegalovirus (HCMV) is indicated in APS, through a peptide of ULB0-HCMVA that shares structural similarity with the phospholipid-binding site of β2GPI [156]. The peptide LKTPRV that is bound by the anti-β2GPI antibodies shows sequence similarity to HCMV [145]. Similarly, human immunodeficiency virus (HIV) shows similarity to the peptide TLRVYK that is recognized by anti-β2GPI antibodies [145]. Finally, hepatitis C virus (HCV) produces a mimotope of the target for RF on IgG1Fc [157].

### 2.5 Retroviral elements

A special situation pose human endogenous retroviruses (=HERVs), which are retroviral elements that permanently integrated into the human genome a long time ago [[158], [159], [160], [161]]. Although mostly non-functional anymore [162], these elements can still produce proteins, such as Gag, Env, or Nef, that are recognized by antiviral immune responses in ARD patients, potentially becoming targets of autoreactivity. HERV-K10 has been implicated as a potential trigger of RA through molecular mimicry [58,157]. Elevated levels of HERV-K10 mRNA have been observed in RA patients [163,164], along with increased reactivity to its Gag proteins [163]. Bioinformatic analysis has identified two peptides from HERV-K10 as mimotopes of IgG1Fc [58], a target of RF, with RA patients showing heightened antibody responses to one of these peptides, further supporting the role of molecular mimicry.

### 2.6 Concluding remarks

To find further microbes of the gut and oral microbiome displaying molecular mimicry to type XI collagen or SE, Zhang *et al.* [121] published an extensive list including metagenomic data in their supplementary material. Moreover, a detailed list of both pathogenic and commensal bacteria, fungi and viruses that could trigger molecular mimicry to various autoantigens can be found in Ref. [55]. More implications of HERVs in RA are discussed in Refs. [157,165].

## 3 Molecular mimicry in systemic lupus erythematosus

SLE is a chronic, multisystem autoimmune disease that can affect many organs, including the joints, skin, central nervous system, and kidneys [166]. It is relatively rare, affecting approximately 0.1 % of the population with a strong gender bias towards women, and manifests heterogeneously [166]. Disease subsets include cutaneous lupus erythematosus, lupus nephritis (which significantly decreases life expectancy through serious complications such as renal failure), and drug-induced SLE [166]. The disease is characterized by high autoantibody titres, with some of the most specific for SLE being anti-Smith (Sm; a component of the spliceosome), anti-Ro60 (RNA binding protein), anti-double-stranded DNA (dsDNA) antibodies [122,166], or anti-ribonucleoprotein (RNP; an RNA-protein complex involved in RNA metabolism) antibodies. The Sm proteins are components of the spliceosome involved in the splicing of nuclear RNAs, and major Sm targets in SLE include Sm B/B’, DI, D2, and D3.

SLE originates from both genetic and environmental factors. Genetic risk factors are various, including mutations within the complement pathway, nucleases, the type I interferon (IFN) pathway or certain high-risk HLA alleles (HLA-DR3 and HLA-DR15) [167]. Environmental triggers include microbial infection, for example through molecular mimicry. The following sections describe several pathobionts, as well as pathogens where molecular mimicry is suggested. Specific mimotopes and autoantigens and, if known, their amino acid sequences, associated in SLE are listed in Table 2. Moreover, Fig. 3 gives an overview of the microbes discussed below.

**Table 2:** **Instances of molecular mimicry between microbial mimotopes and autoantigens in systemic lupus erythematosus (SLE).** This table highlights examples discussed in this review where molecular mimicry has been implicated in SLE pathogenesis. Only microbes are included where both the microbial structure (mimotope) and human autoantigen have been identified, and where some form of cross-reactivity has been observed: ‘Level of evidence’ indicates which cross-reactive component of the adaptive immune system recognizes the mimotope or microbe. While not exhaustive, this list provides a representative overview of the most well-established connections between microbial infections and autoimmune responses in SLE.

Microbe	Microbial structure (mimotope)	Human structure	Level of evidence	Reference
*Actinetobacter johnsonii*	aldehyde dehydrogenase (DKFLEMAVERVKRIK at position 315–329)	Ro60	Murine T-cell hybridomas	[168]
*Actinomyces massiliensis*	Ro60 (RRTMLAVDVSGSMTM)	Ro60 T-cell epitope (KRFLAAVDVSASMNQ at amino acid position 369–383)	T-cells in anti-Ro-60-positive patient	[167]
	Ro60 (KARVHPVSVLVAQRTYAGGA)	Ro60 T-cell epitope (KARIHPFHILIALETYKTGH at amino acid position 316–335)	T-cells (predicted)	[167]
	Ro60 (VKYRQREGWTHRDLLRLAHPRT)	Ro60 B-cell epitope (TKYKQRNGWSHKDLLRLSHLKP at amino acid position 169–190)	B-cells (predicted)	[167]
*Akkermansia muciniphila*	mimotope (DGQFCM)	Fas (DGQFCH)	Antibodies in SLE patients	[169]
*Alistipes finegoldii*	Mg-chelatase subunit ChID (IDIMLAIDVSGSMLA at position 88–102)	Ro60	Murine T-cell hybridomas	[168]
*Bacteroides finegoldii*	vWFA (IDIMLAVDVSTSMLA at amino acid position 88–102)	Ro60	Murine T-cell hybridomas	[168]
*Bacteroides fragilis*	ubiquitin (BfUbb) (KQMEDGRT)	Ubiquitin (KQLEDGRTL)	Antibodies in rabbits	[93,120]
	ubiquitin (BfUbb) (QDKEGFPPDKIRL)	Ubiquitin (QDKEGIPPDQQRL)	Antibodies in rabbits	[93]
	BatA (IDIMLAIDVSTSMLA at amino acid position 88–102)	Ro60	Murine T-cell hybridomas	[168]
*Bacteroides intestinalis*	vWFA (IDIMLAVDVSTSMLA at amino acid position 88–102)	Ro60	Murine T-cell hybridomas	[168]
*Bacteroides thetaiotaomicron*	Ro60	Ro60 (amino acid position 316–335)	B-cells (predicted)	[167]
	Ro60 (TRVLIACDVSGSMQC)	Ro60 T-cell epitope (KRFLAAVDVSASMNQ at amino acid position 369–383)	T-cells of anti-Ro-60-positive patient	[167]
*Capnocytophaga ochracea*	vWFA (IDIVMAIDVSASMLS at amino acid position 92–106)	Ro60	Murine T-cell hybridomas	[168]
*Capnocytopliaga sputigena*	vWFA (IDIVMAIDVSSSMLS at amino acid position 92–106)	Ro60	Murine T-cell hybridomas	[168]
*Corynebacterium amycolatum*	Ro60 (KRTLLSLDVSASMHW)	Ro60 T-cell epitope (KRFLAAVDVSASMNQ at amino acid position 369–383)	T-cells of anti-Ro-60-positive patient	[167]
	Ro60 (VKYRNRGGWSHRDLLRLAHPST)	Ro60 B-cell epitope (TKYKQRNGWSHKDLLRLSHLKP at amino acid position 169–190)	B-cells (predicted)	[167]
	Ro60 (NARIHPVSVLAAQRTYAKGR)	Ro60 T-cell epitope (KARIHPFHILIALETYKTGH at amino acid position 316–335)	B-cells (predicted)	[167]
	TROVE domain protein (KRTLLSLDVSASMHW at amino acid position 366–380)	Ro60	Murine T-cell hybridomas	[168]
Coxsackie virus	Coxsackie virus 2B protein (pepCoxs) (MVTSTITEKL LKNLVKI at position 31–47)	222-229 AA of Ro60 (216KALSVETEKLLKYLEAV232)	Antibodies in SLE patients	[170]
Epstein-Barr virus	EBNA-1 peptide (PPPGRRP)	dsDNA	Antibodies in rabbit	[171]
	EBNA-1 (whole protein)	dsDNA	Antibodies in mice	[172]
	EBNA-1 peptide (AGAGGGAGGAGAGGGAGGAGC)	p542 peptide (GGGGGGSGGGGSGGGGGGGSS)	Antibodies in mice	[173]
	EBNA-1 peptide (GGSGSGPRHRDGVRR at position 58–72)	Ro60 (TKYKQRNGWSHK at position 169–180)	B-cells (predicted)	[49]
	EBNA-1	Sm	Antibodies in mice	[172]
	EBNA-1 peptide (PPPGRRP)	Sm B/B′ peptide (PPPGMRPP)	Antibodies in rabbits	[171]
	EBNA-1 peptide (PPPGRRP)	Sm D	Antibodies in rabbit	[171]
	EBNA-1 peptide with ((GR)X) sequence (GPAGPRGGGRGRGRGRGRGHNDGG at position 35–58)	Sm D1 with with ((GR)X) sequence (RRPGGRGRGRGRGRGRGRGRGRGA at position 95–119)	Antibodies in patient and mice	[174]
	EBNA-3C (PRHGHSQGPWKPWSA at position 886–900)	HTLV-1-related endogenous sequence (HRES-1)/p28 (PRHRHPQDPRSPGPA at position 41–55)	Antibodies in HRES-1/p28-positive lupus patients	[175]
	EBV-LF3	HTLV-1-related endogenous sequence (HRES-1)/p28 (PRHRHPQDPRSPGPA at position 41–55)	Antibodies in HRES-1/p28-positive lupus patients	[175]
*Escherichia coli*	aquaporin	aquaporin 4	Serum in patients, antibodies and T-cells in mice	[176]
Human cytomegalovirus	ULB0-HCMVA (TIFILFCCSKEKRKKKQAAT at position 101–120)	β2GPI (GDKVSFFCKNKEKKC at position 274–288)	Antibodies in mice	[156]
	HCMVpp65	dsDNA	Antibodies in mice	[177]
*Listeria grayi*	Beta lactamase (VIVGQLIEEHRLRYD) at amion acid position 121–135)	Ro60	Murine T-cell hybridomas	[168]
*Mycobacterium tuberculosis*	glycolipids of the cell wall	ssDNA	Antibodies in patients and mice	[178,179]
	Hsp70	DNA	Antibodies in patients	[180]
*Odoribacter splanchnicus*	IS66 family transposase (YLYDGRIFI)	Sm B'B (ILQDGRIFI)	T-cells in patients	[169]
Parvovirus B19	parvovirus VP1 protein peptide (TWGYGSNFGGAV)	cytokeratin (GGGYGSSFGGVD at position 66–77)	Not specified	[146]
	Virus protein 1 and/or 2 (overlapping sequence TWGYGSNFGGAV)	keratin, collagen type II, ssDNA and cardiolipin	Antibodies in patients	[148]
*Prevotella disiens*	vWFA (INIMLAVDISASMLS at amino acid position 88–102)	Ro60	Murine T-cell hybridomas	[168]
*Propionibacterium propionicum*	Ro60 (KRTMLALDVSGSMCS)	Ro60 T-cell epitope (KRFLAAVDVSASMNQ at amino acid position 369–383)	T-cells of anti-Ro-60-positive patient	[167]
	Ro60 (RARVHPVNVLVAGRTYASGR)	Ro60 T-cell epitope (KARIHPFHILIALETYKTGH at amino acid position 316–335)	B-cells	[167]
	Ro60 (VKYRQRSGWTHRDLLRLSHPVT)	Ro60 B-cell epitope (TKYKQRNGWSHKDLLRLSHLKP at amino acid position 169–190)	B-cells (predicted)	[167]
Pseudomonas mendocina	vWFA (RDLLLAVDVSGSMAY at amino acid position 90–104)	Ro60	Murine T-cell hybridomas	[168]
*Roseburia intestinalis*	DNA methyltransferase	β2-glycoprotein I (β2GPI)	Antibodies and T-cells in patients and mice	[122]
*Ruminococcus gnavus*	cell wall lipoglycans	anti-dsDNA	Antibodies in patients	[181]
Transfusion-trasmitted virus	ORF2a	HTLV-1-related endogenous sequence (HRES-1)/p28	Antibodies in patients	[182]

### 3.1 Gut microbiome

The gut commensal *Bacteroides thetaiotaomicron* is increased in patients with SLE [183], especially in those with active disease [184]. Greiling *et al.* [167] showed in an impressive paper that B- and T-cell responses to human Ro60 (hRo60) and lupus-like disease occur after mono-colonization with this bacterium in mice. This finding is supported by evidence that the mimotope in bacterial Ro60 (bRo60) stimulates hRo60-reactive T-cells isolated from SLE patients [167], suggesting molecular mimicry [44,167,185]. Specifically, T-cells reacting to the *B. thetaiotaomicron* mimotope show cross-reactivity to the whole hRo60 or the pathogenic T-cell peptide p316-335, which is known to initiate epitope spreading (see 5.3 Other mechanisms of pathogenicity) in mice [167,186]. Vice versa, hRo60-reactive T-cells proliferated strongly in response to the bRo60 as well as to whole, heat-killed *B. thetaiotaomicron*. Moreover, sequence similarity between another T-cell epitope, the hRo60 peptide 369–383, and bRo60 was observed.

Moreover, *B. fragilis* produces proteins with mimotopes to SLE autoantigens. Namely, it produces BfUbb that mimics human ubiquitin and that is recognized by antibodies recognizing human ubiquitin as already described (see 2.1 Gut microbiome) [93,120]. Furthermore, Szymula *et al.* showed that a peptide of the BatA protein of *B. fragilis* acts as a mimotope of hRo60 and can activate hRo60-reactive T-cells *in vitro* [44,168], though its reactivity *in vivo* has yet to be determined. The same holds true for the species *Bacteroides intestinalis* and *Bacteroides finegoldii* which produce mimotopes in the vWFA protein [168]. A mimotope with similar properties is the Mg-chelatase subunit ChID which contains a cross-reactive sequence and is produced by *Alistipes finegoldii* [44,168]. The *alistipes* genus was found to be more prevalent in SLE patients [184].

The gut microbe *Rumicococcus gnavus* is more abundant in SLE patients [181], especially in females or those with active disease [184]. Additionally, higher antibody titres against it have been observed in SLE patients [44,187]. The strain RG2 produces cell wall lipoglycans that promote the production of cross-reactive anti-dsDNA antibodies of autoreactive B-cells [[181], [187], [188]], indicating molecular mimicry. Another gut microbe, *K. pneumoniae*, which may also turn pathogenic in other parts of the body, has been implicated in the development of anti-dsDNA antibodies. Sera from patients with *K. pneumoniae* infections contain higher titres of anti-dsDNA IgG [189,190], likely due to molecular mimicry [18,158].

Other noteworthy bacteria include the following*: R. intestinalis* expresses a protein, DNA cytosine methyltransferase, that contains a mimotope of B- and T-cell epitopes in β2GPI (see 2.1 Gut microbiome) [122,187]. Antiphospholipid antibodies such as anti-β2GPI are present in up to 40 % of SLE patients, and at least half of these develop APS [191]. *R. intestinalis* is also increased in lupus-induced mice [177]. Another bacterium, *Akkermansia muciniphila,* is enriched in SLE patients and its abundance correlates with inflammation [169]. *A. muciniphila* produces a mimotope of the Fas autoepitope, which is bound by IgG in SLE patients but not in healthy controls [169]. *Odoribacter splanchnicus*, which is reduced in SLE patients after treatment, produces a peptide of the IS66 family transposase that mimics the human Sm B/B’ epitope and that induces the production of pro-inflammatory cytokines in anti-Sm-positive SLE patients [169]. Finally, *E. coli* produces a bacterial aquaporin [192] that cross-reacts with human aquaporin 4, an autoantigen implicated in vision loss in SLE patients [193].

### 3.2 Oral, skin and vaginal microbiome

In the oral microbiome, several bacteria have been implicated in molecular mimicry. *Propionibacterium propionicum* (also known as *Arachnia propionica* or *Pseudopropionibacterium propionicum*) is a commensal found in the oral and skin microbiomes [167]. As other bacteria described above, *P. propionicum* produces bRo60, a mimotope of hRo60 [44,167,185], with T-cells reactive to bRo60 also cross-reacting with hRo60 [167]. Conversely, anti-hRo60 T-cell lines proliferate in response to bRo60 or whole *P. propionicum*. Sera from anti-hRo60-positive SLE patients, but not from anti-hRo60-negative controls, preferentially co-immunoprecipitated bRo60 from *P. propionicum*. Moreover, the human B-cell epitope at amino acid position 169–190 shows sequence similarity to an epitope produced by this commensal. While several epitopes of hRo60 are recognized in patients with SLE, the B-cell epitope 169–190 is the first and most commonly detected [167].

Another oral commensal, *Actinomyces massiliensis*, produces the mimotope bRo60, leading to pathogenic T-cell and autoantibody responses against hRo60 [167]. *A. massiliensis* is enriched in SLE patients and reduced after treatment [169]. Studies show it relocates to the gut in individuals with SLE [185,187].

Szymula *et al.* [168] demonstrated that the oral microbe *Capnocytophaga ochracea* produces a peptide of the vWFA protein acting as a mimotope of hRo60, which was the strongest activator of hRo60-reactive T-cells among 22 peptides tested. Moreover, *Capnocytopliaga sputigena*, part of the normal oral flora and an opportunistic pathogen, and *Prevotella disiens*, part of the oral and vaginal microbiome and associated with bacterial vaginosis, produce vWFA that contains a mimotope activating hRo60-reactive T-cell hybridomas [168]. Whether these bacteria can also activate hRo60-reactive T-cells *in vivo* is an important question to answer.

Members of the skin microbiome also produce mimotopes of hRo60. *Corynebacterium amycolatum* produces bRo60 with three mimotopes of hRo60 [167,185,187], leading to pathogenic T-cell and autoantibody responses. hRo60-reactive T-cells were shown to proliferate in response to this bRo60. One of the three mimotopes discovered by Greiling *et al.* [167] was also identified by Szymula *et al.* [168] to be part of the TROVE domain that activates hRo60-reactive T-cell hybridomas. *Actinetobacter johnsonii*, another skin microbiome member, produces a hRo60 orthologue, the aldehyde dehydrogenase, which also activates hRo60-reactive T-cell hybridomas [168].

### 3.3 Pathogens

Numerous pathogens have been implicated in the aetiology of SLE through molecular mimicry, with viruses providing the most substantial evidence: Studies have demonstrated that EBV load is 10- to 100-fold higher in the peripheral blood of SLE patients compared to controls [48,194,195]. Additionally, SLE patients exhibit an increased prevalence of anti-EBV humoral responses targeting nuclear (EBNA), viral capsid, and early antigens [159,196]. A correlation has been found between the frequency of EBV-infected cells and disease activity [194].

Several instances of cross-reactivity between the EBV protein EBNA-1 and self-peptides have been documented [33,48,142,159,173]. EBNA-1 contains a mimotope that cross-reacts with a peptide of the autoantigen hRo60, which is known to induce lupus [49]. Antibodies against hRo60 also recognize the EBNA-1 peptide, and immunization of rabbits with either peptide leads to the development of cross-reactive antibodies targeting both structures, accompanied by clinical signs of SLE and subsequent epitope spreading. Initially, several Ro60 epitopes are recognized, followed by additional antigens such as Sm B∗, rRNP, and dsDNA. Importantly, EBV infection and antibodies to EBNA-1 have been observed to occur before or at the onset of autoantibodies to Ro, and anti-EBNA-1 antibodies in healthy individuals rarely cross-react with Ro60 or induce autoimmunity, indicating a SLE-specific mechanism of molecular mimicry for EBNA-1 [49].

Regarding Sm autoantigens, EBNA-1 contains a mimotope of Sm B/B′ and the amino acid sequence PPPGMRPP of Sm D [171]. Antibodies from SLE patients commonly recognize Sm sequences, while anti-EBV-positive healthy (non-SLE) subjects do not. The amino acid sequence PPPGMRPP is suggested to be the initial epitope recognized by Sm B′-specific autoantibodies [197], potentially triggering Sm B′ autoimmunity in SLE patients through molecular mimicry with EBNA-1 [49]. Immunization with the EBNA-1 peptide in rabbits induces antibodies against Sm D and Sm B/B’ [198,199]. A longer EBNA-1 peptide with a ((GR)X) repeat has been observed to mimic a Sm D1 sequence with a similar repeat [174], with antibodies from SLE patients binding to these cross-reactive molecules. Similarly, mice immunized with the EBNA-1 sequence also develop antibodies targeting both peptides. Another study showed that mice develop antibodies to Sm after injection with a vector containing the entire EBNA-1 molecule [172].

Double-stranded DNA (dsDNA) represents another category of autoantigens with mimotopes derived from EBV. In an older study, immunization with the EBNA-1 peptide PPPGRRP [171] induced anti-dsDNA antibodies in one out of two rabbits. Although the sample size is too small for definitive conclusions, a subsequent study with a larger sample size produced similar results: In this case, immunization with a vector encoding the entire EBNA-1 antigen led to the development of anti-dsDNA antibodies in ten mice [172]. Moreover, the autoantigen p542, a target of IgG autoantibodies in SLE patients, has a epitope (glycine repeat) cross-reactive with the EBNA-1 peptide, and anti-p542 antibodies also bind EBNA-1, further supporting the role of molecular mimicry in SLE [173].

Other viruses implicated in SLE aetiology include PB19 [146], HCMV [156], HPV [200] and coxsackie virus [170]. Anti-viral IgG antibodies from patients with persistent PB19 infection cross-react with several autoantigens [148], such as ss-DNA and cardiolipin [148]. Additionally, PB19 infection increases the levels of antinuclear antibodies, anti-dsDNA, anti-LA-SSB, anticardiolipin, and anti-β2-glycoprotein I antibodies [201]. A mimotope of the PB19 VP1 protein shares similarity with the human cytokeratin [146], and anti-VP1 and anti-VP2 IgG antibodies from PB19-infected patients specifically react with human keratin, collagen type II, ssDNA, and cardiolipin [148].

HCMV is indicated in APS, which is associated with SLE, through molecular mimicry. A mimotope of ULB0-HCMVA shares structural similarity with the phospholipid-binding site of β2GPI [156], and the peptide LKTPRV that is bound by anti-β2GPI antibodies shows similarity to HCMV antigens [145]. Moreover, the viral peptide HCMVpp65 was shown to trigger lupus in mice via the generation of anti-dsDNA autoantibodies [169]. Mice immunized with the viral peptide generated antiphospholipid antibodies, and their activity was inhibited by incubation with cardiolipin liposomes and β2GPI [156]. Moreover, the antibodies were shown to be pathogenic in mice *in vivo* [156], overall suggesting that molecular may be the mechanism through which pathogenic antiphospholipid antibodies are generated [156,158].

For HPV, structural protein sequences of high-risk types were analysed for sequence similarity to human proteins implicated in SLE [200]. 13 peptides were identified to be 100 % identical to human peptides and predicted to bind to HLA alleles, mainly to the SLE-predisposing DRB1 allele [200]. Moreover, coxsackie virus has been implicated, with a mimotope of its 2B protein (pepCoxs) showing 87 % amino acid similarity to a major antibody-binding site on hRo60 [170]. SLE patients reacting to the hRo60 peptide sequence also react to the pepCoxs sequence, suggesting that the virus plays a role in the formation of anti-Ro autoantibodies.

Moreover, several bacterial pathogens, such as *Mycobacterium tuberculosis*, the causative agent of tuberculosis, have been reported to be potentially implicated in SLE through molecular mimicry [158,188]. Patients with pulmonary tuberculosis exhibit increased levels of anti-dsDNA, anti-ssDNA, anti-polynucleotide and anti-cardiolipin antibodies [178]. Antibodies against *M. tuberculosis* glycolipids, components of the mycobacterial cell wall, cross-react with ssDNA and other polynucleotides. Vice versa, human anti-DNA antibodies bind to the bacterial glycolipids, indicating molecular mimicry [179]. Additionally, cross-reactivity between Hsp70 and IgG autoantibodies targeting anti-DNA from SLE patients [180], as well as between mycobacterial aquaporins and human aquaporin 4 [176], has been observed.

Moreover, *Pseudomonas mendocina* and *Listeria grayi*, two opportunistic pathogens, produce hRo60 mimotopes (in vWFA and β-lactamase, respectively) that activate hRo60-reactive T-cell hybridomas *in vitro* [168]. Another homology to Ro/SSA is observed in a nucleocapsid protein of vesicular stomatitis virus (VSV), a rhabdovirus, suggesting that infection with VSV could induce anti-Ro humoral responses [202]. *Streptococcus pneumoniae* has also been implicated, with antibodies from patients with lupus binding both pneumococcal polysaccharide and dsDNA, indicating cross-reactivity [203]. Finally, it could be predicted that *Clostridium botulinum* produces an extensive list of proteins that share significant similarities with human proteins, suggesting potential mimicry [204].

One noteworthy parasite is *Leishmania* sp., the causative agent of leishmaniasis, which can present with SLE-like syndromes [205]. Patients with leishmaniasis show cross-reactive antibodies against nuclear antigens and conserved proteins in *Leishmania*. Several cross-reactive autoantigens have been predicted bioinformatically, including Sm D1, Sm B, BiP, DNA topoisomerase, and aquaporin-4 [54].

### 3.4 Retroviral elements

A notable example of a HERV implicated in SLE is the HTLV-1-related endogenous sequence (HRES-1), recognized by antibodies in 52 % of SLE patients. Anti-HRES-1 antibodies are particularly present in patients who do not possess anti-Ro and anti-La antibodies [206]. HRES-1 produces a 28-kDa nuclear autoantigen HRES-1/p28, which shares sequence identity with the 70-kDa gag-related region of the U1 sn-RNP protein. This instance of molecular mimicry may initiate the production of autoantibodies, mediate epitope spreading, and further contribute to the formation of antinuclear autoantibodies in SLE [206].

Moreover, HRES-1 is implicated in cross-reactivity with infectious exogenous retroviruses. Specifically, there is cross-reactivity between the HRES-1/p28 protein and human T-cell lymphotropic virus (HTLV-1) and HIV Gag proteins, which are detected by cross-reactive antibodies in SLE patients [207]. This could also explain why approximately one-third of SLE patients produce antibodies to HIV, even though no prior exposure or infection was observed [160]. Additionally, it is noteworthy that HIV shows similarity to the peptide TLRVYK that is recognized by anti-β2GPI antibodies [145]. Cross-reactivity has also been observed with an ORF2a peptide of transfusion-transmitted virus (TTV), which is found at elevated levels in SLE patients [161,175]. The majority of these patients recognize this peptide, suggesting a potential mechanism for autoimmunity. Generally, all HRES-1/p28-positive SLE patients recognize at least one TTV peptide [175]. HRES-1/p28 also contains mimotopes to EBV antigens, specifically EBV-LF3 and EBNA-3C [182]. This cross-reactivity may contribute to the generation of antinuclear antibodies, abnormal T- and B-cell functions, and self-reactivity in SLE [161,175]. Thus, HERV-encoded proteins such as HRES-1 can be viewed to occupy a unique position in autoimmunity. They are thought to act either as foreign mimics of self-peptides (e.g., HRES-1/p28 mimicking U1 sn-RNP) or as autoantigens themselves, triggering immune responses in SLE patients. This dual role suggests that HERVs could be key players in the development of autoimmune diseases.

### 3.5 Concluding remarks

Further microbiota that are activating hRo60-reactive T-cells *in vitro* can be found in Ref. [168]. Further HPV peptides with sequence similarity to human peptides are predicted in Ref. [200]. More predictions of molecular mimicry between antigens from *Leishmania* sp. and autoantigens can be found in Ref. [54].

## 4 Molecular mimicry in other ARDs

Other ARDs, including AS, SSc, and myositis, have fewer documented instances of molecular mimicry compared to RA and SLE. This raises the question of whether molecular mimicry plays a smaller role in these diseases, or if other factors contribute to the scarcity of data. One possible factor is the prevalence of these ARDs, as higher patient numbers increase the likelihood of uncovering such associations. RA (0.5–1 % [16]) and SLE (approximately 0.1 % [166]) are more common than SSc (approximately 0.02 % [208]) and myositis (approximately 0.02–0.06 % [209,210]), which may explain the fewer associations identified for the latter two. AS, with a prevalence of approximately 0.18 % [211], is similarly common to SLE. However, it is underrepresented in terms of publications available, both in absolute numbers and in relation to its prevalence. These disparities could be the reason why fewer instances of molecular mimicry are observed for AS, SSc and myositis. Nevertheless, the following paragraphs summarize the limited data available for these diseases.

The disparities in prevalence and publication numbers in both absolute and relative terms are illustrated in Supplementary Fig. 1. However, these numbers should only be seen as estimates, as different studies report varying prevalence rates, and data is especially limited for the rarer diseases.

### 4.1 Ankylosing spondylitis

AS is a relatively rare ARD that primarily affects the lumbar spine and sacroiliac joints [212]. A strong genetic association with the (class I) HLA-B27 allele has been established [212,213]. Several theories on the pathogenic role of HLA-B27 have been proposed [214], including molecular mimicry by microbes [155], but clarity is still lacking. The microbiome is implicated in AS, as germ-free mice do not develop spondyloarthropathies [215], and microbial dysbiosis has been observed in AS patients [216].

One promising candidate for molecular mimicry in AS is *K. pneumoniae*. AS patients have increased antibody titres against this bacterium [217] and specifically also against its nitrogenase enzyme, particularly during active disease phases [218,219]. This enzyme shares the amino acid sequence QTDRED with HLA-B27 [219]. Moreover, antisera directed against synthetic peptides of this enzyme also react with synovial biopsies from HLA-B27-positive AS patients but not with those from HLA-B27-negative RA patients [218]. Elevated antibody levels against *K. pneumoniae* nitrogenase reductase have also been observed in AS patients [220].

Furthermore, *K. pneumoniae* produces pullulanase, which contains mimotopes of types I, III and IV collagen [221]. Antibody levels in AS patients were elevated against pullulanase and types I and IV collagen compared to controls [221]. Since these collagen types are present in the spine, this could explain the spinal lesions characteristic of AS [221]. Moreover, anti-microbial antibodies induced by *Klebsiella* have been found to bind HLA molecules on lymphocytes, fibroblasts, and chondrocytes, especially in HLA-B27-positive patients [216,[222], [223], [224]]. Collectively, these studies indicate that molecular mimicry with *Klebsiella*, especially with *K. pneumoniae*, could induce autoantibodies against HLA-B27 and spinal collagens.

Other microbes, specifically *Enterobacter aerogenes*, *K. pneumoniae*, *Yersinia enterocolitica* and *Shigella sonnei* strains, were shown to bind to antibodies from one or two rabbits previously immunized with HLA-B27-positive lymphocytes, indicating that these microbes might also be involved in molecular mimicry in AS [225]. However, this hypothesis requires validation in studies with larger sample sizes to draw more definite conclusions.

*Y. enterocolitica* and *Y. pseudotuberculosis* were shown to produce the outer membrane protein yersinia adhesin (YadA), and *Salmonella typhimurium* the outer membrane protein OmpH, both of which share an amino acid sequence with HLA-B27 [226,227]. The shared amino acid sequence in YadA is also part of *K. pneumoniae* nitrogenase [226]. Antibody binding against peptides corresponding to the YadA and OmpH was observed in one-third of HLA-B27-associated diseases [226]. These microbes are promising candidates for further research to determine their potential involvement in molecular mimicry in AS.

### 4.2 Systemic sclerosis

SSc is an autoimmune connective tissue disorder characterized by massive fibrosis, vascular damage, dysregulation of adaptive and innate immunity, and the aberrant activation of fibrotic signalling pathways affecting the skin and internal organs [228,229]. SSc-specific autoantigens include centromere protein (CENP)-A or -B [57], topoisomerase I and fibrillarin, a 34-kDa nucleolar protein associated with U3-snRNP [230].

One promising viral agent associated with SSc is HCMV [53,231,232]. Antibodies specific for the autoantigen topoisomerase I bind to a sequence similar to the UL70 protein of HCMV, suggesting the activation of autoreactive B-cell clones by molecular mimicry [233]. Additionally, the viral UL94 mimotope shows sequence similarity to NAG-2, a cell surface molecule highly expressed on non-stressed endothelial cells and associated with integrins. Anti-UL94 peptide antibodies induce apoptosis of endothelial cells upon engagement with the NAG-2-integrin complex and activate fibroblasts, potentially triggering the hallmarks of SSc, vascular damage and fibrosis, through molecular mimicry [234,235].

As with other ARDs, EBV is also implicated in SSc. EBNA-1 shows sequence similarity to CENP-A and -B [236]. Additionally, fibrillarin shares sequence similarity with an EBV-encoded nuclear protein [236] and the P40 protein expressed by herpes simplex virus (HSV) type I [230]. This indicates that molecular mimicry might significantly contribute to autoimmunity and the production of autoantibodies reacting with CENP-A/B and fibrillarin. *Helicobacter pylori*-associated Hsp60, which shares high sequence similarity with human Hsp60, is found in increased amounts in SSc patients [[237], [238], [239]]. *K. pneumoniae*, also implicated in RA, SLE and AS, was bioinformatically predicted to produce a partial histone H3, a mimotope of the CENP-A protein [53]. Other precited microbial mimotopes of CENP-A or topoisomerase I are listed by Gkoutzourelas *et al.* [53].

Furthermore, it was bioinformatically predicted that viruses of the mimiviridae and phycodnaviridae families show sequence similarity to topoisomerase I, fibrillarin, and CENP-A [57]. Although these viruses do not directly infect humans, they could theoretically still be presented to our immune system indirectly via infected amoeba or algae. Additionally, case reports of SSc development following SARS-CoV-2 infection suggest that this virus could also trigger molecular mimicry in this disease [240,241]. Further research is warranted to substantiate these indications of molecular mimicry in SSc.

### 4.3 Myositis

Myositis refers to a heterogenous group of ARDs characterized by muscle weakness caused by chronic inflammation of the muscles, and various other clinical manifestations [242]. Other affected organs can be the lung, heart, gastrointestinal tract or skin. When the skin is involved, presenting with rashes, the condition is classified as dermatomyositis (DM).

Generally, microbial organisms are known to cause infective myositis [243]. The potential role of microbes in the aetiology of myositis through molecular mimicry remains vastly unexplored. For DM, a single paper has researched the potential role of molecular mimicryby identifying autoantibody responses in patients against 12 different TRIM proteins, including TRIM33, TRIM3 and TRIM47. They demonstrated sequence similarity between TRIM3 epitopes and variola virus, as well as an epitope of TRIM47 and HIV-1 and *Synechococcus* phage syn9, implicating molecular mimicry in DM pathogenesis [244].

## 5 Outlook

This review provides a comprehensive overview of molecular mimicry in RA and SLE, while also briefly considering other ARDs such as AS, SSc, and myositis. It highlights the involvement of various microbiome components - including the gut, oral, urogenital, respiratory, and skin microbiota - as well as pathogens and HERVs, in ARD pathology through molecular mimicry. While some microbes are known to induce autoimmunity, the exact mimotopes are still unidentified in many cases [74]. For other microbes, mimotopes have been identified but clinical data is still lacking [55]. Nevertheless, growing evidence links the concept of molecular mimicry with autoimmune disease aetiology, with significant implications for disease outcomes.

### 5.1 The role of different microbe populations

In RA and SLE, the gut microbiome and pathogens appear to be the primary sources of molecular mimicry. The gut microbiome is more implicated in RA, with *P. copri* and *P. mirabilis* as key players, while pathogens, especially viruses such as EBV and HCMV, are particularly significant in SLE. The oral microbiome, notably *P. gingivalis*, also plays a crucial role in RA by triggering the generation of ACPAs. However, there are notable gaps in understanding the roles of the skin, urogenital, respiratory and especially the vaginal microbiome, in ARDs. An astonishingly low number of reports addresses the potential involvement of the vaginal microbiome in these diseases, despite a female:male ratio of 9:1 in SLE [245] and 3:1 in RA [246,247] and SSc [248]. A PubMed Search with the query “Vaginal Microbiome Molecular Mimicry” (August 23rd, 2024) yielded three results, out of which only one was relevant and only briefly mentioned vaginal involvement [168].

Most microbes implicated in RA and SLE differ, likely due to distinct autoantibody profiles (ACPA and RF in RA vs. anti-Sm and anti-Ro60 in SLE). However, some microbes such as *R. intestinalis*, *K. pneumoniae*, EBV, and HCMV are shared between these conditions. This overlap may be attributed to the presence of overlapping autoantibody profiles, such as anti-β2GPI in RA and SLE, or collagen in RA, SLE and AS, and the pathogenic versatility of these microbes. EBV exemplifies this, being linked to several autoimmune diseases, including multiple sclerosis [249,250], via a variety of mimotopes. Similarly, *K. pneumoniae* is remarkably versatile, producing mimotopes of several different autoantigens and being involved in RA, SLE, AS and SSc.

Given that only a subset of patients with these diseases develops autoantibodies against specific autoantigens (e.g., 30–50 % of SLE patients against Ro [251]), different microbes may trigger disease in different subgroups, highlighting the need for personalized approaches in studying and treating ARDs.

### 5.2 Clinical implications

Understanding the role of microbes in ARD pathogenesis opens new avenues for clinical applications. For instance, vaccines targeting key pathogens such as EBV, HCMV, or *P. gingivalis* could potentially prevent or mitigate these diseases [[252], [253], [254]]. Moreover, modifying the microbiome to decrease the abundance of pathobionts that mimic self-antigens, while promoting beneficial microbes, presents a promising therapeutic strategy [255]. Examples of beneficial microbes are *Helicobacter pylori* and *Toxoplasma gondii*, with antibodies to the former being decreased in SLE patients [256], and infection with the latter inhibiting the development of lupus-like syndrome in mice [257]. Furthermore, *Dialister* and *Haemophilus* species are reduced in SLE [258] and RA patients [121], respectively. Another potential approach includes rebalancing the altered *Firmicutes*/*Bacteroidetes* ratio observed in SLE patients [183]. Faecal microbiota transplantation (FMT) [[259], [260]] and next-generation probiotics [[261], [262]] offer viable options, though challenges such as stable colonization remain [263]. Additionally, targeted antibiotic or antiviral therapies could be considered, though their use must be carefully balanced against the risk of disrupting beneficial bacteria [264].

### 5.3 Other mechanisms of pathogenicity

While this review focuses on molecular mimicry, it is important to acknowledge other mechanisms of pathogenicity contributing to ARD development. These include microbial translocation, where microbes move from their usual sites to other organs, inducing inflammation. For instance, in the context of SLE, *Enterococcus gallinarum* and *R. gnavus* have been observed to translocate from the gut to the liver [265] and lung [266], respectively, while *A. massiliensis* relocates from the oral to the gut microbiome [185,169,267].

Another significant mechanism is epitope spreading, where an immune response initially targets a specific antigen but later expands to recognize further epitopes. This process is well-documented in RA and SLE, contributing to disease progression. For example, hRo60 epitope spreading in SLE is linked to commensals such as *P. propionicum*, *C. amycolatum*, and *A. massiliensis* [167]. Moreover, microbes from the oral microbiome, such as *P. gingivalis*, *V. parvula* or different *Streptococcus* sp. can become citrullinated, triggering autoantibody responses that extend to human citrullinated epitopes in RA [132]. Also, HCMV [121], HCV [268] and PB19 [269] are implicated in epitope spreading in SLE [270].

Bystander activation, where non-specific immune responses during infections inadvertently activate autoreactive T-cells, is another critical factor in autoimmunity [270]. This mechanism has been observed after HCV infection [271], and in RA through bacterial lipopolysaccharide (LPS) produced by bacteria [272]. Finally, the polarization of gut-associated T-cells into pathogenic subsets or activation of T-cells co-expressing dual (autoreactive and microbial-specific) TCRs represents another route through which microbial infections may drive autoimmunity [17].

## CRediT authorship contribution statement

**Michaela Fehringer:** Writing – review & editing, Writing – original draft, Visualization, Conceptualization. **Thomas Vogl:** Writing – review & editing, Supervision, Project administration, Funding acquisition, Conceptualization.

## Declaration of generative AI and AI-assisted technologies in the writing process

During the preparation of this work the first author used ChatGPT in order to improve linguistic clarity and flow. After using this tool/service, the authors reviewed and edited the content as needed and take full responsibility for the content of the publication.

## Funding sources

This work was supported by the Austrian Science Fund (Grant-DOI: 10.55776/DOC188) and 10.13039/100018693Horizon Europe (Project ID: 101136582).

**Table:** Glossary

**Abbreviation**	**Meaning**
ACPA	Anti-citrullinated protein antibodies
APS	Antiphospholipid syndrome
ARD	Autoimmune rheumatoid disease
AS	Ankylosing spondylitis
BCR	B-cell receptor
BfUbb	B. fragilis ubiquitin
BiP	Binding immunoglobulin protein
CENP	Centromeric protein
DM	Dermatomyositis
DR1	HLA-DRB1∗01
DR4	HLA-DRB1∗04
dsDNA	double-stranded DNA
EBNA-1	EBV nuclear antigen-1
EBV	Epstein-Barr virus
FMT	Faecal microbiota transplantation
HCMV	Human cytomegalovirus
HCV	Hepatitis C virus
HERV	Human endogenous retrovirus
HHV	Human herpes virus
HIV	Human immunodeficiency virus
Hpm B	Hemolysin B
HPV	Human papillomavirus
HRES-1	HTLV-1-related endogenous sequence
hRo60	human Ro60
Hsp	Heat shock protein
HSV	Herpes simplex virus
IFN	Interferon
Ig	Immunoglobulin
MHC	Major histocompatibility complex
PAD	Peptidylarginin-deiminase
PB19	Parvovirus B19
pepCoxs	Coxsackie virus 2B protein
PSRA	Post-streptococcal reactive arthritis
RA	Rheumatoid arthritis
RF	Rheumatoid factor
RNP	ribonucleoprotein
SE	Shared epitope
SLE	Systemic lupus erythematosus
Sm	Smith
SSc	Systemic Sclerosis
TCR	T-cell receptor
TTV	Transfusion-transmitted virus
UreC	Urease C
VSV	Vesicular stomatitis virus
YadA	Yersinia adhesin
β2GPI	β2 glycoprotein I

## Declaration of competing interest

The authors declare that they have no known competing financial interests or personal relationships that could have appeared to influence the work reported in this paper.

## Data Availability

No data was used for the research described in the article.

## References

[1] Moutsopoulos H.M. (2021). Autoimmune rheumatic diseases: one or many diseases?. J. Transl. Autoimmun..

[2] El-Shebiny E.M., Zahran E.S., Shoeib S.A., Habib E.S. (2021). Bridging autoinflammatory and autoimmune diseases, Egypt. J. Intern. Med..

[3] Hedar A.M., Stradner M.H., Roessler A., Goswami N. (2021). Autoimmune rheumatic diseases and vascular function: the concept of autoimmune atherosclerosis.

[4] Miller F.W. (2022). The increasing prevalence of autoimmunity and autoimmune diseases: an urgent call to action for improved understanding, diagnosis, treatment and prevention. Curr. Opin. Immunol..

[5] Pisetsky D.S. (2023). Pathogenesis of autoimmune disease. Nat. Rev. Nephrol..

[6] Ramos P.S., Shedlock A.M., Langefeld C.D. (2015). Genetics of autoimmune diseases: insights from population genetics. J. Hum. Genet..

[7] Harroud A., Hafler D.A. (2023). Common genetic factors among autoimmune diseases. Science.

[8] Cotsapas C., Hafler D.A. (2013). Immune-mediated disease genetics: the shared basis of pathogenesis. Trends Immunol.

[9] Oliver J.E., Silman A.J. (2009). Why are women predisposed to autoimmune rheumatic diseases?. Arthritis Res. Ther..

[10] Zheng Y., Liu Q., Goronzy J.J., Weyand C.M. (2023). Immune aging – a mechanism in autoimmune disease. Semin. Immunol..

[11] Goronzy J.J., Weyand C.M. (2012). Immune aging and autoimmunity. Cell. Mol. Life Sci. CMLS.

[12] Bach J.-F. (2018). The hygiene hypothesis in autoimmunity: the role of pathogens and commensals. Nat. Rev. Immunol..

[13] Bach J.-F. (2002). The effect of infections on susceptibility to autoimmune and allergic diseases. N. Engl. J. Med..

[14] Procaccini C., Carbone F., Galgani M., La Rocca C., De Rosa V., Cassano S., Matares G. (2011). Obesity and susceptibility to autoimmune diseases. Expert Rev. Clin. Immunol..

[15] Perricone C., Versini M., Ben-Ami D., Gertel S., Watad A., Segel M.J., Ceccarelli F., Conti F., Cantarini L., Bogdanos D.P., Antonelli A., Amital H., Valesini G., Shoenfeld Y. (2016). Smoke and autoimmunity: the fire behind the disease. Autoimmun. Rev..

[16] Smolen J.S., Aletaha D., Barton A., Burmester G.R., Emery P., Firestein G.S., Kavanaugh A., McInnes I.B., Solomon D.H., Strand V., Yamamoto K. (2018). Rheumatoid arthritis. Nat. Rev. Dis. Primer.

[17] Garabatos N., Santamaria P. (2022). Gut microbial antigenic mimicry in autoimmunity. Front. Immunol..

[18] Rashid T., Ebringer A. (2012). Autoimmunity in rheumatic diseases is induced by microbial infections via crossreactivity or molecular mimicry. Autoimmune Dis..

[19] Kriegel M.A. (2023). Subdoligranulum chews up joints: how a gut pathobiont can instigate arthritis. Trends Immunol..

[20] Charles A Janeway J., Travers P., Walport M., Shlomchik M.J. (2001). Immunobiol. Immune Syst. Health Dis.

[21] Pishesha N., Harmand T.J., Ploegh H.L. (2022). A guide to antigen processing and presentation. Nat. Rev. Immunol..

[22] Wildner G. (2023). Antigenic mimicry – the key to autoimmunity in immune privileged organs. J. Autoimmun..

[23] Chi X., Li Y., Qiu X. (2020). V(D)J recombination, somatic hypermutation and class switch recombination of immunoglobulins: mechanism and regulation. Immunology.

[24] Nishana M., Raghavan S.C. (2012). Role of recombination activating genes in the generation of antigen receptor diversity and beyond. Immunology.

[25] Sewell A.K. (2012). Why must T cells be cross-reactive?. Nat. Rev. Immunol..

[26] Mason D. (1998). A very high level of crossreactivity is an essential feature of the T-cell receptor. Immunol. Today.

[27] Arstila T.P., Casrouge A., Baron V., Even J., Kanellopoulos J., Kourilsky P. (1999). A direct estimate of the human alphabeta T cell receptor diversity. Science.

[28] Wooldridge L., Ekeruche-Makinde J., van den Berg H.A., Skowera A., Miles J.J., Tan M.P., Dolton G., Clement M., Llewellyn-Lacey S., Price D.A., Peakman M., Sewell A.K. (2012). A single autoimmune T cell receptor recognizes more than a million different peptides. J. Biol. Chem..

[29] Abbas A.K., Lichtman A.H., Pillai S. (2021). https://shop.elsevier.com/books/cellular-and-molecular-immunology/abbas/978-0-323-75748-5.

[30] Charles A Janeway J., Travers P., Walport M., Shlomchik M.J. (2001). Immunobiol. Immune Syst. Health Dis.

[31] Fairlie-Clarke K.J., Shuker D.M., Graham A.L. (2009). Why do adaptive immune responses cross-react?. Evol. Appl..

[32] Liang B., Mamula M.J. (2000). Molecular mimicry and the role of B lymphocytes in the processing of autoantigens. Cell. Mol. Life Sci. CMLS.

[33] Suliman B.A. (2024). Potential clinical implications of molecular mimicry-induced autoimmunity. Immun. Inflamm. Dis..

[34] Rojas M., Restrepo-Jiménez P., Monsalve D.M., Pacheco Y., Acosta-Ampudia Y., Ramírez-Santana C., Leung P.S.C., Ansari A.A., Gershwin M.E., Anaya J.-M. (2018). Molecular mimicry and autoimmunity. J. Autoimmun..

[35] Ursell L.K., Metcalf J.L., Parfrey L.W., Knight R. (2012). Defining the human microbiome. Nutr. Rev..

[36] Sender R., Fuchs S., Milo R. (2016). Revised estimates for the number of human and bacteria cells in the body. PLoS Biol.

[37] Sr G., Rt D., Pb E., Pj T., Bs S., Ji G., Da R., Cm F.-L., Ke N. (2006). Metagenomic analysis of the human distal gut microbiome. Science.

[38] Turnbaugh P.J., Ley R.E., Mahowald M.A., Magrini V., Mardis E.R., Gordon J.I. (2006). An obesity-associated gut microbiome with increased capacity for energy harvest. Nature.

[39] Bäckhed F., Ding H., Wang T., Hooper L.V., Koh G.Y., Nagy A., Semenkovich C.F., Gordon J.I. (2004). The gut microbiota as an environmental factor that regulates fat storage. Proc. Natl. Acad. Sci. U. S. A..

[40] Bäckhed F., Manchester J.K., Semenkovich C.F., Gordon J.I. (2007). Mechanisms underlying the resistance to diet-induced obesity in germ-free mice. Proc. Natl. Acad. Sci. U. S. A..

[41] Martin F.-P.J., Dumas M.-E., Wang Y., Legido-Quigley C., Yap I.K.S., Tang H., Zirah S., Murphy G.M., Cloarec O., Lindon J.C., Sprenger N., Fay L.B., Kochhar S., van Bladeren P., Holmes E., Nicholson J.K. (2007). A top-down systems biology view of microbiome-mammalian metabolic interactions in a mouse model. Mol. Syst. Biol..

[42] Mazmanian S.K., Liu C.H., Tzianabos A.O., Kasper D.L. (2005). An immunomodulatory molecule of symbiotic bacteria directs maturation of the host immune system. Cell.

[43] Hooper L.V., Stappenbeck T.S., Hong C.V., Gordon J.I. (2003). Angiogenins: a new class of microbicidal proteins involved in innate immunity. Nat. Immunol..

[44] English J., Patrick S., Stewart L.D. (2023). The potential role of molecular mimicry by the anaerobic microbiota in the aetiology of autoimmune disease. Anaerobe.

[45] Lin L., Zhang K., Xiong Q., Zhang J., Cai B., Huang Z., Yang B., Wei B., Chen J., Niu Q. (2023). Gut microbiota in pre-clinical rheumatoid arthritis: from pathogenesis to preventing progression. J. Autoimmun..

[46] Chow J., Mazmanian S.K. (2010). A pathobiont of the microbiota balances host colonization and intestinal inflammation. Cell Host Microbe.

[47] Mazmanian S.K., Round J.L., Kasper D.L. (2008). A microbial symbiosis factor prevents intestinal inflammatory disease. Nature.

[48] Toussirot É., Roudier J. (2008). Epstein–Barr virus in autoimmune diseases. Best Pract. Res. Clin. Rheumatol..

[49] McClain M.T., Heinlen L.D., Dennis G.J., Roebuck J., Harley J.B., James J.A. (2005). Early events in lupus humoral autoimmunity suggest initiation through molecular mimicry. Nat. Med..

[50] Roudier J., Petersen J., Rhodes G.H., Luka J., Carson D.A. (1989). Susceptibility to rheumatoid arthritis maps to a T-cell epitope shared by the HLA-Dw4 DR beta-1 chain and the Epstein-Barr virus glycoprotein gp110. Proc. Natl. Acad. Sci. U. S. A..

[51] Baboonian C., Halliday D., Venables P.J., Pawlowski T., Millman G., Maini R.N. (1989). Antibodies in rheumatoid arthritis react specifically with the glycine alanine repeat sequence of Epstein-Barr nuclear antigen-1. Rheumatol. Int..

[52] van Eden W., Tholet J.E.R., van der Zee R., Noordzij A., van Embden J.D.A., Hensen E.J., Cohen I.R. (1988). Cloning of the mycobacterial epitope recognized by T lymphocytes in adjuvant arthritis. Nature.

[53] Gkoutzourelas A., Barmakoudi M., Bogdanos D.P. (2020). A bioinformatics analysis reveals novel pathogens as molecular mimicry triggers of systemic sclerosis, mediterr. J. Rheumatol..

[54] Múnera M., Farak J., Pérez M., Rojas J., Villero J., Sánchez A., Sánchez J., Emiliani Y. (2020). Prediction of molecular mimicry between antigens from Leishmania sp. and human: implications for autoimmune response in systemic lupus erythematosus. Microb. Pathog..

[55] Repac J., Mandić M., Lunić T., Božić B., Nedeljković B.B. (2021). Mining the capacity of human-associated microorganisms to trigger rheumatoid arthritis—a systematic immunoinformatics analysis of T cell epitopes. PLoS One.

[56] Karami Fath M., Jahangiri A., Ganji M., Sefid F., Payandeh Z., Hashemi Z.S., Pourzardosht N., Hessami A., Mard-Soltani M., Zakeri A., Rahbar M.R., Khalili S. (2021). SARS-CoV-2 proteome harbors peptides which are able to trigger autoimmunity responses: implications for infection, vaccination, and population coverage. Front. Immunol..

[57] Gourh P., Safran S.A., Alexander T., Boyden S.E., Morgan N.D., Shah A.A., Mayes M.D., Doumatey A., Bentley A.R., Shriner D., Domsic R.T., Medsger T.A., Ramos P.S., Silver R.M., Steen V.D., Varga J., Hsu V., Saketkoo L.A., Schiopu E., Khanna D., Gordon J.K., Kron B., Criswell L.A., Gladue H., Derk C.T., Bernstein E.J., Bridges S.L., Shanmugam V.K., Kolstad K.D., Chung L., Kafaja S., Jan R., Trojanowski M., Goldberg A., Korman B.D., Steinbach P.J., Chandrasekharappa S.C., Mullikin J.C., Adeyemo A., Rotimi C., Wigley F.M., Kastner D.L., Boin F., Remmers E.F. (2020). HLA and autoantibodies define scleroderma subtypes and risk in African and European Americans and suggest a role for molecular mimicry. Proc. Natl. Acad. Sci. U. S. A..

[58] Nelson P.N., Roden D., Nevill A., Freimanis G.L., Trela M., Ejtehadi H.D., Bowman S., Axford J., Veitch A.M., Tugnet N., Rylance P.B. (2014). Rheumatoid arthritis is associated with IgG antibodies to human endogenous retrovirus gag matrix: a potential pathogenic mechanism of disease?. J. Rheumatol..

[59] Altschul S.F., Gish W., Miller W., Myers E.W., Lipman D.J. (1990). Basic local alignment search tool. J. Mol. Biol..

[60] Sayers E.W., Beck J., Bolton E.E., Bourexis D., Brister J.R., Canese K., Comeau D.C., Funk K., Kim S., Klimke W., Marchler-Bauer A., Landrum M., Lathrop S., Lu Z., Madden T.L., O’Leary N., Phan L., Rangwala S.H., Schneider V.A., Skripchenko Y., Wang J., Ye J., Trawick B.W., Pruitt K.D., Sherry S.T. (2020). Database resources of the national center for Biotechnology information. Nucleic Acids Res..

[61] The UniProt Consortium (2023). UniProt: the universal protein knowledgebase in 2023. Nucleic Acids Res..

[62] Yan Z., Kim K., Kim H., Ha B., Gambiez A., Bennett J., de Almeida Mendes M.F., Trevizani R., Mahita J., Richardson E., Marrama D., Blazeska N., Koşaloğlu-Yalçın Z., Nielsen M., Sette A., Peters B., Greenbaum J.A. (2024). Next-generation IEDB tools: a platform for epitope prediction and analysis. Nucleic Acids Res..

[63] Lundegaard C., Lamberth K., Harndahl M., Buus S., Lund O., Nielsen M. (2008). NetMHC-3.0: accurate web accessible predictions of human, mouse and monkey MHC class I affinities for peptides of length 8-11. Nucleic Acids Res..

[64] Jurtz V., Paul S., Andreatta M., Marcatili P., Peters B., Nielsen M. (2017). NetMHCpan 4.0: improved peptide-MHC class I interaction predictions integrating eluted ligand and peptide binding affinity data. J. Immunol. Baltim..

[65] Waterhouse A., Bertoni M., Bienert S., Studer G., Tauriello G., Gumienny R., Heer F.T., de Beer T.A.P., Rempfer C., Bordoli L., Lepore R., Schwede T. (2018). SWISS-MODEL: homology modelling of protein structures and complexes. Nucleic Acids Res..

[66] Jumper J., Evans R., Pritzel A., Green T., Figurnov M., Ronneberger O., Tunyasuvunakool K., Bates R., Žídek A., Potapenko A., Bridgland A., Meyer C., Kohl S.A.A., Ballard A.J., Cowie A., Romera-Paredes B., Nikolov S., Jain R., Adler J., Back T., Petersen S., Reiman D., Clancy E., Zielinski M., Steinegger M., Pacholska M., Berghammer T., Bodenstein S., Silver D., Vinyals O., Senior A.W., Kavukcuoglu K., Kohli P., Hassabis D. (2021). Highly accurate protein structure prediction with AlphaFold. Nature.

[67] Chastain D.B., Stover K.R. (2023). Urgent need to address infectious diseases due to immunosuppressive therapies. Ther. Adv. Infect. Dis..

[68] Knight K.L., Burnett R.C., McNicholas J.M. (1950). Organization and polymorphism of rabbit immunoglobulin heavy chain genes. J. Immunol. Baltim. Md.

[69] Almagro J.C., Hernández I., Ramírez M.C., Vargas-Madrazo E. (1998). Structural differences between the repertoires of mouse and human germline genes and their evolutionary implications. Immunogenetics.

[70] Smolen J.S., Aletaha D., McInnes I.B. (2016). Rheumatoid arthritis. Lancet Lond. Engl..

[71] Kim J.-W., Suh C.-H. (2020). Systemic manifestations and complications in patients with rheumatoid arthritis. J. Clin. Med..

[72] Costenbader K.H., Karlson E.W. (2006). Epstein–Barr virus and rheumatoid arthritis: is there a link?. Arthritis Res. Ther..

[73] Maibom-Thomsen S.L., Trier N.H., Holm B.E., Hansen K.B., Rasmussen M.I., Chailyan A., Marcatili P., Højrup P., Houen G. (2019). Immunoglobulin G structure and rheumatoid factor epitopes. PLoS One.

[74] Chriswell M.E., Lefferts A.R., Clay M.R., Hsu A.R., Seifert J., Feser M.L., Rims C., Bloom M.S., Bemis E.A., Liu S., Maerz M.D., Frank D.N., Demoruelle M.K., Deane K.D., James E.A., Buckner J.H., Robinson W.H., Holers V.M., Kuhn K.A. (2022). Clonal IgA and IgG autoantibodies from individuals at risk for rheumatoid arthritis identify an arthritogenic strain of Subdoligranulum. Sci. Transl. Med..

[75] Lu D.R., McDavid A.N., Kongpachith S., Lingampalli N., Glanville J., Ju C.-H., Gottardo R., Robinson W.H. (2018). T cell-dependent affinity maturation and innate immune pathways differentially drive autoreactive B cell responses in rheumatoid arthritis. Arthritis Rheumatol. Hoboken NJ.

[76] van Zeben D., Hazes J.M., Zwinderman A.H., Cats A., van der Voort E.A., Breedveld F.C. (1992). Clinical significance of rheumatoid factors in early rheumatoid arthritis: results of a follow up study. Ann. Rheum. Dis..

[77] Curran A.M., Naik P., Giles J.T., Darrah E. (2020). PAD enzymes in rheumatoid arthritis: pathogenic effectors and autoimmune targets. Nat. Rev. Rheumatol..

[78] Auger I., Sebbag M., Vincent C., Balandraud N., Guis S., Nogueira L., Svensson B., Cantagrel A., Serre G., Roudier J. (2005). Influence of HLA–DR genes on the production of rheumatoid arthritis–specific autoantibodies to citrullinated fibrinogen. Arthritis Rheum..

[79] Nogueira N., Sebbag M., Chapuy-Regaud S., Clavel C., Fournie B., Cantagrel A., Vincent C., Serre G. (2002). Autoantibodies to deiminated fibrinogen are the most efficient serological criterion for the diagnosis of rheumatoid arthritis. Arthritis Res. Ther..

[80] Corrigall V.M., Panayi G.S. (2002). Autoantigens and immune pathways in rheumatoid arthritis. Crit. Rev. Immunol..

[81] Tong D., Lönnblom E., Yau A.C.Y., Nandakumar K.S., Liang B., Ge C., Viljanen J., Li L., Bãlan M., Klareskog L., Chagin A.S., Gjertsson I., Kihlberg J., Zhao M., Holmdahl R. (2018). A shared epitope of collagen type XI and type II is recognized by pathogenic antibodies in mice and humans with arthritis. Front. Immunol..

[82] Mantej J., Polasik K., Piotrowska E., Tukaj S. (2018). Autoantibodies to heat shock proteins 60, 70, and 90 in patients with rheumatoid arthritis. Cell Stress Chaperones.

[83] Corrigall V.M., Bodman-Smith M.D., Fife M.S., Canas B., Myers L.K., Wooley P., Soh C., Staines N.A., Pappin D.J., Berlo S.E., van Eden W., van Der Zee R., Lanchbury J.S., Panayi G.S. (2001). The human endoplasmic reticulum molecular chaperone BiP is an autoantigen for rheumatoid arthritis and prevents the induction of experimental arthritis. J. Immunol. Baltim. Md.

[84] Melayah S., Ghozzi M., Mankaï A., Mechi F., Ghedira I. (2022). Frequency of serological markers of rheumatoid arthritis in patients with IgA anti‐β2 glycoprotein I antibodies. J. Clin. Lab. Anal..

[85] Berryman M.A., Ilonen J., Triplett E.W., Ludvigsson J. (2023). Important denominator between autoimmune comorbidities: a review of class II HLA, autoimmune disease, and the gut. Front. Immunol..

[86] Gregersen P.K., Silver J., Winchester R.J. (1987). The shared epitope hypothesis. an approach to understanding the molecular genetics of susceptibility to rheumatoid arthritis. Arthritis Rheum..

[87] Wysocki T., Olesińska M., Paradowska-Gorycka A. (2020). Current understanding of an emerging role of HLA-DRB1 gene in rheumatoid arthritis–from research to clinical practice. Cells.

[88] Zhuo J., Xia Q., Sharma N., Gao S., Lama S., Cui J., Feathers V., Shadick N., Weinblatt M.E. (2022). The role of shared epitope in rheumatoid arthritis prognosis in relation to anti-citrullinated protein antibody positivity. Rheumatol. Ther.

[89] Roudier J., Balandraud N., Auger I. (2022). How RA associated HLA-DR molecules contribute to the development of antibodies to citrullinated proteins: the hapten carrier model. Front. Immunol..

[90] Trier N., Izarzugaza J., Chailyan A., Marcatili P., Houen G. (2018). Human MHC-II with shared epitope motifs are optimal epstein-barr virus glycoprotein 42 ligands—relation to rheumatoid arthritis. Int. J. Mol. Sci..

[91] Hill J.A., Southwood S., Sette A., Jevnikar A.M., Bell D.A., Cairns E. (1950). Cutting edge: the conversion of arginine to citrulline allows for a high-affinity peptide interaction with the rheumatoid arthritis-associated HLA-DRB1∗0401 MHC class II molecule. J. Immunol. Baltim. Md.

[92] Auger I., Balandraud N., Massy E., Hemon M.F., Peen E., Arnoux F., Mariot C., Martin M., Lafforgue P., Busnel J.-M., Roudier J. (2020). Peptidylarginine deiminase autoimmunity and the development of anti-citrullinated protein antibody in rheumatoid arthritis: the hapten-carrier model. Arthritis Rheumatol. Hoboken NJ.

[93] Stewart L., Edgar J.D.M., Blakely G., Patrick S. (2018). Antigenic mimicry of ubiquitin by the gut bacterium Bacteroides fragilis: a potential link with autoimmune disease. Clin. Exp. Immunol..

[94] Cornillet M., Verrouil E., Cantagrel A., Serre G., Nogueira L. (2015). ACPA-positive RA patients, antibodies to EBNA35-58Cit, a citrullinated peptide from the Epstein–Barr nuclear antigen-1, strongly cross-react with the peptide β60-74Cit which bears the immunodominant epitope of citrullinated fibrin. Immunol. Res..

[95] Birkenfeld P., Haratz N., Klein G., Sulitzeanu D. (1990). Cross-reactivity between the EBNA-1 p107 peptide, collagen, and keratin: implications for the pathogenesis of rheumatoid arthritis. Clin. Immunol. Immunopathol..

[96] Baboonian C., Venables P.J., Williams D.G., Williams R.O., Maini R.N. (1991). Cross reaction of antibodies to a glycine/alanine repeat sequence of Epstein-Barr virus nuclear antigen-1 with collagen, cytokeratin, and actin. Ann. Rheum. Dis..

[97] Moore K.W., Vieira P., Fiorentino D.F., Trounstine M.L., Khan T.A., Mosmann T.R. (1990). Homology of cytokine synthesis inhibitory factor (IL-10) to the Epstein-Barr virus gene BCRFI. Science.

[98] Sin S.-H., Dittmer D.P. (2012). Cytokine homologs of human gammaherpesviruses. J. Interferon Cytokine Res..

[99] Vieira P., de Waal-Malefyt R., Dang M.N., Johnson K.E., Kastelein R., Fiorentino D.F., deVries J.E., Roncarolo M.G., Mosmann T.R., Moore K.W. (1991). Isolation and expression of human cytokine synthesis inhibitory factor cDNA clones: homology to Epstein-Barr virus open reading frame BCRFI. Proc. Natl. Acad. Sci. U. S. A..

[100] Albani S., Keystone E.C., Nelson J.L., Ollier W.E.R., La Cava A., Montemayor A.C., Weber D.A., Montecucco C., Martini A., Carson D.A. (1995). Positive selection in autoimmunity: abnormal immune responses to a bacterial dnaJ antigenic determinant in patients with early rheumatoid arthritis. Nat. Med..

[101] Moten D., Teneva I., Apostolova D., Batsalova T., Dzhambazov B. (2022). Molecular mimicry of the rheumatoid arthritis-related immunodominant T-cell epitope within type II collagen (CII260-270) by the bacterial L-asparaginase. Int. J. Mol. Sci..

[102] Lee J.Y., Choi I.A., Kim J.-H., Kim K.-H., Lee E.Y., Lee E.B., Lee Y.-M., Song Y.W. (2015). Association between anti-Porphyromonas gingivalis or anti-α-enolase antibody and severity of periodontitis or rheumatoid arthritis (RA) disease activity in RA. BMC Musculoskelet. Disord..

[103] Kinloch A.J., Alzabin S., Brintnell W., Wilson E., Barra L., Wegner N., Bell D.A., Cairns E., Venables P.J. (2011). Immunization with Porphyromonas gingivalis enolase induces autoimmunity to mammalian α-enolase and arthritis in DR4-IE–transgenic mice. Arthritis Rheum..

[104] Pianta A., Arvikar S.L., Strle K., Drouin E.E., Wang Q., Costello C.E., Steere A.C. (2017). Two rheumatoid arthritis–specific autoantigens correlate microbial immunity with autoimmune responses in joints. J. Clin. Invest..

[105] Ebringer A., Cunningham P., Ahmadi K., Wrigglesworth J., Hosseini R., Wilson C. (1992). Sequence similarity between HLA-DR1 and DR4 subtypes associated with rheumatoid arthritis and proteus/serratia membrane haemolysins. Ann. Rheum. Dis..

[106] Wilson C., Ebringer A., Ahmadi K., Wrigglesworth J., Tiwana H., Fielder M., Binder A., Ettelaie C., Cunningham P., Joannou C. (1995). Shared amino acid sequences between major histocompatibility complex class II glycoproteins, type XI collagen and Proteus mirabilis in rheumatoid arthritis. Ann. Rheum. Dis..

[107] Wilson C., Tiwana H., Ebringer A., Cinningham P., Ettelaie C. (1997). HLA-DR4 restriction, molecular mimicry and rheumatoid arthritis. Immunol. Today.

[108] Tett A., Huang K.D., Asnicar F., Fehlner-Peach H., Pasolli E., Karcher N., Armanini F., Manghi P., Bonham K., Zolfo M., De Filippis F., Magnabosco C., Bonneau R., Lusingu J., Amuasi J., Reinhard K., Rattei T., Boulund F., Engstrand L., Zink A., Collado M.C., Littman D.R., Eibach D., Ercolini D., Rota-Stabelli O., Huttenhower C., Maixner F., Segata N. (2019). The Prevotella copri complex comprises four distinct clades underrepresented in westernized populations. Cell Host Microbe.

[109] Alpizar-Rodriguez D., Lesker T.R., Gronow A., Gilbert B., Raemy E., Lamacchia C., Gabay C., Finckh A., Strowig T. (2019). Prevotella copri in individuals at risk for rheumatoid arthritis. Ann. Rheum. Dis..

[110] Scher J.U., Sczesnak A., Longman R.S., Segata N., Ubeda C., Bielski C., Rostron T., Cerundolo V., Pamer E.G., Abramson S.B., Huttenhower C., Littman D.R. (2013). Expansion of intestinal Prevotella copri correlates with enhanced susceptibility to arthritis. Elife.

[111] Maeda Y., Takeda K. (2017). Role of gut microbiota in rheumatoid arthritis. J. Clin. Med..

[112] Maeda Y., Kurakawa T., Umemoto E., Motooka D., Ito Y., Gotoh K., Hirota K., Matsushita M., Furuta Y., Narazaki M., Sakaguchi N., Kayama H., Nakamura S., Iida T., Saeki Y., Kumanogoh A., Sakaguchi S., Takeda K. (2016). Dysbiosis contributes to arthritis development via activation of autoreactive T cells in the intestine. Arthritis Rheumatol. Hoboken NJ.

[113] Pianta A., Arvikar S., Strle K., Drouin E.E., Wang Q., Costello C.E., Steere A.C. (2017). Evidence of the immune relevance of Prevotella copri, a gut microbe, in patients with rheumatoid arthritis. Arthritis Rheumatol. Hoboken NJ.

[114] Pianta A., Chiumento G., Ramsden K., Wang Q., Strle K., Arvikar S., Costello C.E., Steere A.C. (2021). Identification of novel, immunogenic HLA-DR-presented Prevotella copri peptides in patients with rheumatoid arthritis. Arthritis Rheumatol. Hoboken NJ.

[115] Seifert J.A., Bemis E.A., Ramsden K., Lowell C., Polinski K., Feser M., Fleischer C., Demoruelle M.K., Buckner J., Gregersen P.K., Keating R.M., Mikuls T.R., O’Dell J.R., Weisman M.H., Deane K.D., Norris J.M., Steere A.C., Holers V.M. (2023). Association of antibodies to Prevotella copri in anti–cyclic citrullinated peptide-positive individuals at risk of developing rheumatoid arthritis and in patients with early or established rheumatoid arthritis. Arthritis Rheumatol..

[116] Ebringer A., Rashid T. (2006). Rheumatoid arthritis is an autoimmune disease triggered by *Proteus* urinary tract infection. J. Immunol. Res..

[117] Frank J.F., Hassan A.N., Roginski H. (2002). Encycl. Dairy Sci..

[118] Christopoulos G., Christopoulou V., Routsias J.G., Babionitakis A., Antoniadis C., Vaiopoulos G. (2017). Greek rheumatoid arthritis patients have elevated levels of antibodies against antigens from Proteus mirabilis. Clin. Rheumatol..

[119] Ebringer A., Rashid T., Wilson C. (2010). Rheumatoid arthritis, Proteus, anti-CCP antibodies and Karl Popper. Autoimmun. Rev..

[120] Patrick S., Jobling K.L., O’Connor D., Thacker Z., Dryden D.T.F., Blakely G.W. (2011). A unique homologue of the eukaryotic protein-modifier ubiquitin present in the bacterium Bacteroides fragilis, a predominant resident of the human gastrointestinal tract. Microbiology.

[121] Zhang X., Zhang D., Jia H., Feng Q., Wang D., Liang D., Wu X., Li J., Tang L., Li Y., Lan Z., Chen B., Li Y., Zhong H., Xie H., Jie Z., Chen W., Tang S., Xu X., Wang X., Cai X., Liu S., Xia Y., Li J., Qiao X., Al-Aama J.Y., Chen H., Wang L., Wu Q., Zhang F., Zheng W., Li Y., Zhang M., Luo G., Xue W., Xiao L., Li J., Chen W., Xu X., Yin Y., Yang H., Wang J., Kristiansen K., Liu L., Li T., Huang Q., Li Y., Wang J. (2015). The oral and gut microbiomes are perturbed in rheumatoid arthritis and partly normalized after treatment. Nat. Med..

[122] Ruff W.E., Dehner C., Kim W.J., Pagovich O., Aguiar C.L., Yu A.T., Roth A.S., Vieira S.M., Kriegel C., Adeniyi O., Mulla M.J., Abrahams V.M., Kwok W.W., Nussinov R., Erkan D., Goodman A.L., Kriegel M.A. (2019). Pathogenic autoreactive T and B cells cross-react with mimotopes expressed by a common human gut commensal to trigger autoimmunity. Cell Host Microbe.

[123] Bishu S., Su E.W., Wilkerson E.R., Reckley K.A., Jones D.M., McGeachy M.J., Gaffen S.L., Levesque M.C. (2014). Rheumatoid arthritis patients exhibit impaired Candida albicans-specific Th17 responses. Arthritis Res. Ther..

[124] Alghamdi M.A., Redwan E.M. (2022). Interplay of microbiota and citrullination in the immunopathogenesis of rheumatoid arthritis. Probiotics Antimicrob. Proteins.

[125] Drago L. (2019). Prevotella copri and microbiota in rheumatoid arthritis: fully convincing evidence?. J. Clin. Med..

[126] Lappin D.F., Apatzidou D., Quirke A.-M., Oliver-Bell J., Butcher J.P., Kinane D.F., Riggio M.P., Venables P., McInnes I.B., Culshaw S. (2013). Influence of periodontal disease, Porphyromonas gingivalis and cigarette smoking on systemic anti-citrullinated peptide antibody titres. J. Clin. Periodontol..

[127] Sherina N., de Vries C., Kharlamova N., Sippl N., Jiang X., Brynedal B., Kindstedt E., Hansson M., Mathsson-Alm L., Israelsson L., Stålesen R., Saevarsdottir S., Holmdahl R., Hensvold A., Johannsen G., Eriksson K., Sallusto F., Catrina A.I., Rönnelid J., Grönwall C., Yucel-Lindberg T., Alfredsson L., Klareskog L., Piccoli L., Malmström V., Amara K., Lundberg K. (2022). Antibodies to a citrullinated Porphyromonas gingivalis epitope are increased in early rheumatoid arthritis, and can Be produced by gingival tissue B cells: implications for a bacterial origin in RA etiology. Front. Immunol..

[128] Wegner N., Wait R., Sroka A., Eick S., Nguyen K.-A., Lundberg K., Kinloch A., Culshaw S., Potempa J., Venables P.J. (2010). Peptidylarginine deiminase from Porphyromonas gingivalis citrullinates human fibrinogen and α-enolase: implications for autoimmunity in rheumatoid arthritis. Arthritis Rheum..

[129] de Aquino S.G., Abdollahi-Roodsaz S., Koenders M.I., van de Loo F.A.J., Pruijn G.J.M., Marijnissen R.J., Walgreen B., Helsen M.M., van den Bersselaar L.A., de Molon R.S., Campos M.J.A., Cunha F.Q., Cirelli J.A., van den Berg W.B. (2014). Periodontal pathogens directly promote autoimmune experimental arthritis by inducing a TLR2- and IL-1–driven Th17 response. J. Immunol..

[130] Lundberg K., Kinloch A., Fisher B.A., Wegner N., Wait R., Charles P., Mikuls T.R., Venables P.J. (2008). Antibodies to citrullinated α-enolase peptide 1 are specific for rheumatoid arthritis and cross-react with bacterial enolase. Arthritis Rheum..

[131] Kinloch A.J., Alzabin S., Brintnell W., Wilson E., Barra L., Wegner N., Bell D.A., Cairns E., Venables P.J. (2011). Immunization with Porphyromonas gingivalis enolase induces autoimmunity to mammalian α-enolase and arthritis in DR4-IE-transgenic mice. Arthritis Rheum..

[132] Brewer R.C., Lanz T.V., Hale C.R., Sepich-Poore G.D., Martino C., Swafford A.D., Carroll T.S., Kongpachith S., Blum L.K., Elliott S.E., Blachere N.E., Parveen S., Fak J., Yao V., Troyanskaya O., Frank M.O., Bloom M.S., Jahanbani S., Gomez A.M., Iyer R., Ramadoss N.S., Sharpe O., Chandrasekaran S., Kelmenson L.B., Wang Q., Wong H., Torres H.L., Wiesen M., Graves D.T., Deane K.D., Holers V.M., Knight R., Darnell R.B., Robinson W.H., Orange D.E. (2023). Oral mucosal breaks trigger anti-citrullinated bacterial and human protein antibody responses in rheumatoid arthritis. Sci. Transl. Med..

[133] Ahmed S., Padhan P., Misra R., Danda D. (2021). Update on post-streptococcal reactive arthritis: narrative review of a forgotten disease. Curr. Rheumatol. Rep..

[134] Konig M.F., Abusleme L., Reinholdt J., Palmer R.J., Teles R.P., Sampson K., Rosen A., Nigrovic P.A., Sokolove J., Giles J.T., Moutsopoulos N.M., Andrade F. (2016). Aggregatibacter actinomycetemcomitans-induced hypercitrullination links periodontal infection to autoimmunity in rheumatoid arthritis. Sci. Transl. Med..

[135] Scher J.U., Joshua V., Artacho A., Abdollahi-Roodsaz S., Öckinger J., Kullberg S., Sköld M., Eklund A., Grunewald J., Clemente J.C., Ubeda C., Segal L.N., Catrina A.I. (2016). The lung microbiota in early rheumatoid arthritis and autoimmunity. Microbiome.

[136] Kawahito Y., Ichinose S., Sano H., Tsubouchi Y., Kohno M., Yoshikawa T., Tokunaga D., Hojo T., Harasawa R., Nakano T., Matsuda K. (2008). Mycoplasma fermentans glycolipid-antigen as a pathogen of rheumatoid arthritis. Biochem. Biophys. Res. Commun..

[137] Citti C., Nouvel L.-X., Baranowski E. (2010). Phase and antigenic variation in mycoplasmas. Future Microbiol..

[138] Estevão S., Sluijter M., Hartwig N.G., van Rossum A.M.C., Vink C. (2011). Functional characterization of the RuvB homologs from Mycoplasma pneumoniae and Mycoplasma genitalium. J. Bacteriol..

[139] Qin L., Chen Y., You X. (2019). Subversion of the immune response by human pathogenic mycoplasmas. Front. Microbiol..

[140] Masuoka S., Kusunoki N., Takamatsu R., Takahashi H., Tsuchiya K., Kawai S., Nanki T. (2018). Epstein-Barr virus infection and variants of Epstein-Barr nuclear antigen-1 in synovial tissues of rheumatoid arthritis. PLoS One.

[141] Balandraud N., Meynard J.B., Auger I., Sovran H., Mugnier B., Reviron D., Roudier J., Roudier C. (2003). Epstein-Barr virus load in the peripheral blood of patients with rheumatoid arthritis: accurate quantification using real-time polymerase chain reaction. Arthritis Rheum..

[142] Petersen J., Rhodes G., Roudier J., Vaughan J.H. (1990). Altered immune response to glycine-rich sequences of Epstein-Barr nuclear antigen-1 in patients with rheumatoid arthritis and systemic lupus erythematosus. Arthritis Rheum..

[143] Mangalam A., Luckey D., Basal E., Jackson M., Smart M., Rodriguez M., David C. (2009). HLA-DQ8 (DQB1∗0302) restricted Th17 cells exacerbate experimental autoimmune encephalomyelitis in HLA-DR3 transgenic mice. J. Immunol. Baltim. Md.

[144] Paulsen S.J., Rosenkilde M.M., Eugen-Olsen J., Kledal T.N. (2005). Epstein-barr virus-encoded BILF1 is a constitutively active G protein-coupled receptor. J. Virol..

[145] Blank M., Shoenfeld Y., Cabilly S., Heldman Y., Fridkin M., Katchalski-Katzir E. (1999). Prevention of experimental antiphospholipid syndrome and endothelial cell activation by synthetic peptides. Proc. Natl. Acad. Sci. U. S. A..

[146] Lunardi C., Tinazzi E., Bason C., Dolcino M., Corrocher R., Puccetti A. (2008). Human parvovirus B19 infection and autoimmunity. Autoimmun. Rev..

[147] Mehraein Y., Lennerz C., Ehlhardt S., Venzke T., Ojak A., Remberger K., Zang K.D. (2003). Detection of parvovirus B19 capsid proteins in lymphocytic cells in synovial tissue of autoimmune chronic arthritis. Mod. Pathol..

[148] Lunardi C., Tiso M., Borgato L., Nanni L., Millo R., De Sandre G., Severi A.B., Puccetti A. (1998). Chronic parvovirus B19 infection induces the production of anti-virus antibodies with autoantigen binding properties. Eur. J. Immunol..

[149] Marín J.S., Mazenett-Granados E.A., Salazar-Uribe J.C., Sarmiento M., Suárez J.F., Rojas M., Munera M., Pérez R., Morales C., Dominguez J.I., Anaya J.-M. (2023). Increased incidence of rheumatoid arthritis after COVID-19. Autoimmun. Rev..

[150] Gaston J.S., Life P.F., Jenner P.J., Colston M.J., Bacon P.A. (1990). Recognition of a mycobacteria-specific epitope in the 65-kD heat-shock protein by synovial fluid-derived T cell clones. J. Exp. Med..

[151] Karlsson-Parra A., Söderström K., Ferm M., Ivanyi J., Kiessling R., Klareskog L. (1990). Presence of human 65 kD heat shock protein (hsp) in inflamed joints and subcutaneous nodules of RA patients. Scand. J. Immunol..

[152] Quayle A.J., Wilson K.B., Li S.G., Kjeldsen‐Kragh J., Oftung F., Shinnick T., Sioud M., Førre Ø., Capra J.D., Natvig J.B. (1992). Peptide recognition, T cell receptor usage and HLA restriction elements of human heat‐shock protein (hsp) 60 and mycobacterial 65‐kDa hsp‐reactive T cell clones from rheumatoid synovial fluid. Eur. J. Immunol..

[153] Ogra P.L., Herd J.K. (1971). Arthritis associated with induced rubella Infection1. J. Immunol..

[154] Veetil B.M.A., Myasoedova E., Matteson E.L., Gabriel S.E., Green A.B., Crowson C.S. (2013). Incidence and time trends of herpes zoster in rheumatoid arthritis: a population-based cohort study. Arthritis Care Res..

[155] Wilson C., Tiwana H., Ebringer A. (2000). Molecular mimicry between HLA-DR alleles associated with rheumatoid arthritis and Proteus mirabilis as the aetiological basis for autoimmunity. Microbes Infect.

[156] Gharavi A.E., Pierangeli S.S., Espinola R.G., Liu X., Colden-Stanfield M., Harris E.N. (2002). Antiphospholipid antibodies induced in mice by immunization with a cytomegalovirus-derived peptide cause thrombosis and activation of endothelial cells in vivo. Arthritis Rheum..

[157] Trela M., Nelson P.N., Rylance P.B. (2016). The role of molecular mimicry and other factors in the association of Human Endogenous Retroviruses and autoimmunity. APMIS.

[158] Zandman-Goddard G., Shoenfeld Y., Zandman-Goddard G., Shoenfeld Y., Infections and SLE (2005). Autoimmunity.

[159] Agmon-Levin N., Blank M., Paz Z., Shoenfeld Y. (2009). Molecular mimicry in systemic lupus erythematosus. Lupus.

[160] Adelman M.K., Marchalonis J.J. (2002). Endogenous retroviruses in systemic lupus erythematosus: candidate lupus viruses. Clin. Immunol..

[161] Rigante D., Mazzoni M.B., Esposito S. (2014). The cryptic interplay between systemic lupus erythematosus and infections. Autoimmun. Rev..

[162] Stearrett N., Dawson T., Rahnavard A., Bachali P., Bendall M.L., Zeng C., Caricchio R., Pérez-Losada M., Grammer A.C., Lipsky P.E., Crandall K.A. (2021). Expression of human endogenous retroviruses in systemic lupus erythematosus: multiomic integration with gene expression. Front. Immunol..

[163] Ejtehadi H.D., Freimanis G.L., Ali H.A., Bowman S., Alavi A., Axford J., Callaghan R., Nelson P.N. (2006). The potential role of human endogenous retrovirus K10 in the pathogenesis of rheumatoid arthritis: a preliminary study. Ann. Rheum. Dis..

[164] Reynier F., Verjat T., Turrel F., Imbert P.E., Marotte H., Mougin B., Miossec P. (2009). Increase in human endogenous retrovirus HERV-K (HML-2) viral load in active rheumatoid arthritis. Scand. J. Immunol..

[165] Tugnet N., Rylance P., Roden D., Trela M., Nelson P. (2013). Human endogenous retroviruses (HERVs) and autoimmune rheumatic disease: is there a link?. Open Rheumatol. J..

[166] Kaul A., Gordon C., Crow M.K., Touma Z., Urowitz M.B., van Vollenhoven R., Ruiz-Irastorza G., Hughes G. (2016). Systemic lupus erythematosus. Nat. Rev. Dis. Primer.

[167] Greiling T.M., Dehner C., Chen X., Hughes K., Iñiguez A.J., Boccitto M., Ruiz D.Z., Renfroe S.C., Vieira S.M., Ruff W.E., Sim S., Kriegel C., Glanternik J., Chen X., Girardi M., Degnan P., Costenbader K.H., Goodman A.L., Wolin S.L., Kriegel M.A. (2018). Commensal orthologs of the human autoantigen Ro60 as triggers of autoimmunity in lupus. Sci. Transl. Med..

[168] Szymula A., Rosenthal J., Szczerba B.M., Bagavant H., Fu S.M., Deshmukh U.S. (2014). T cell epitope mimicry between Sjögren’s syndrome Antigen A (SSA)/Ro60 and oral, gut, skin and vaginal bacteria. Clin. Immunol..

[169] Chen B., Jia X., Xu J., Zhao L., Ji J., Wu B., Ma Y., Li H., Zuo X., Pan W., Wang X., Ye S., Tsokos G.C., Wang J., Zhang X. (2021). An autoimmunogenic and proinflammatory profile defined by the gut microbiota of patients with untreated systemic lupus erythematosus. Arthritis Rheumatol..

[170] Stathopoulou E.A., Routsias J.G., Stea E.A., Moutsopoulos H.M., Tzioufas A.G. (2005). Cross-reaction between antibodies to the major epitope of Ro60 kD autoantigen and a homologous peptide of Coxsackie virus 2B protein. Clin. Exp. Immunol..

[171] James J.A., Scofield R.H., Harley J.B. (1997). Lupus humoral autoimmunity after short peptide immunization. Ann. N. Y. Acad. Sci..

[172] Sundar K., Jacques S., Gottlieb P., Villars R., Benito M.-E., Taylor D.K., Spatz L.A. (2004). Expression of the Epstein-Barr virus nuclear antigen-1 (EBNA-1) in the mouse can elicit the production of anti-dsDNA and anti-Sm antibodies. J. Autoimmun..

[173] Vaughan J.H., Nguyen M.D., Valbracht J.R., Patrick K., Rhodes G.H. (1995). Epstein-Barr virus-induced autoimmune responses. II. Immunoglobulin G autoantibodies to mimicking and nonmimicking epitopes. Presence in autoimmune disease. J. Clin. Invest..

[174] Sabbatini A., Bombardieri S., Migliorini P. (1993). Autoantibodies from patients with systemic lupus erythematosus bind a shared sequence of SmD and Epstein-Barr virus-encoded nuclear antigen EBNA I. Eur. J. Immunol..

[175] Gergely P., Pullmann R., Stancato C., Otvos L., Koncz A., Blazsek A., Poor G., Brown K.E., Phillips P.E., Perl A. (2005). Increased prevalence of transfusion-transmitted virus and cross-reactivity with immunodominant epitopes of the HRES-1/p28 endogenous retroviral autoantigen in patients with systemic lupus erythematosus. Clin. Immunol. Orlando Fla.

[176] Cossu D., Yokoyama K., Tomizawa Y., Momotani E., Hattori N. (2017). Altered humoral immunity to mycobacterial antigens in Japanese patients affected by inflammatory demyelinating diseases of the central nervous system. Sci. Rep..

[177] Chen Y.-F., Hsieh A.-H., Wang L.-C., Huang Y.-J., Tsai Y.-C., Tseng W.-Y., Kuo Y.-L., Luo S.-F., Yu K.-H., Kuo C.-F. (2021). Fecal microbiota changes in NZB/W F1 mice after induction of lupus disease. Sci. Rep..

[178] Sela O., el-Roeiy A., Isenberg D.A., Kennedy R.C., Colaco C.B., Pinkhas J., Shoenfeld Y. (1987). A common anti-DNA idiotype in sera of patients with active pulmonary tuberculosis. Arthritis Rheum..

[179] Shoenfeld Y., Vilner Y., Coates A.R., Rauch J., Lavie G., Shaul D., Pinkhas J. (1986). Monoclonal anti-tuberculosis antibodies react with DNA, and monoclonal anti-DNA autoantibodies react with Mycobacterium tuberculosis. Clin. Exp. Immunol..

[180] Tasneem S., Islam N., Ali R. (2001). Crossreactivity of SLE autoantibodies with 70 kDa heat shock proteins of Mycobacterium tuberculosis. Microbiol. Immunol..

[181] Azzouz D., Omarbekova A., Heguy A., Schwudke D., Gisch N., Rovin B.H., Caricchio R., Buyon J.P., Alekseyenko A.V., Silverman G.J. (2019). Lupus nephritis is linked to disease-activity associated expansions and immunity to a gut commensal. Ann. Rheum. Dis..

[182] Perl A., Nagy G., Koncz A., Gergely P., Fernandez D., Doherty E., Telarico T., Bonilla E., Phillips P.E. (2008). Molecular mimicry and immunomodulation by the HRES-1 endogenous retrovirus in SLE. Autoimmunity.

[183] van der Meulen T.A., Harmsen H.J.M., Vila A.V., Kurilshikov A., Liefers S.C., Zhernakova A., Fu J., Wijmenga C., Weersma R.K., de Leeuw K., Bootsma H., Spijkervet F.K.L., Vissink A., Kroese F.G.M. (2019). Shared gut, but distinct oral microbiota composition in primary Sjögren’s syndrome and systemic lupus erythematosus. J. Autoimmun..

[184] Toumi E., Goutorbe B., Plauzolles A., Bonnet M., Mezouar S., Militello M., Mege J.-L., Chiche L., Halfon P. (2022). Gut microbiota in systemic lupus erythematosus patients and lupus mouse model: a cross species comparative analysis for biomarker discovery. Front. Immunol..

[185] Ruff W.E., Greiling T.M., Kriegel M.A. (2020). Host–microbiota interactions in immune-mediated diseases. Nat. Rev. Microbiol..

[186] Deshmukh U.S., Lewis J.E., Gaskin F., Kannapell C.C., Waters S.T., Lou Y., Tung K.S.K., Fu S.M. (1999). Immune responses to Ro60 and its peptides in mice. I. The nature of the immunogen and endogenous autoantigen determine the specificities of the induced autoantibodies. J. Exp. Med..

[187] Redanz S., Kriegel M.A. (2022). The role of the microbiome in lupus and antiphospholipid syndrome. Z. Rheumatol..

[188] Vitale A.M., Paladino L., Caruso Bavisotto C., Barone R., Rappa F., Conway de Macario E., Cappello F., Macario A.J.L., Marino Gammazza A. (2024). Interplay between the chaperone system and gut microbiota dysbiosis in systemic lupus erythematosus pathogenesis: is molecular mimicry the missing link between those two factors?. Int. J. Mol. Sci..

[189] el-Roiey A., Gross W.L., Luedemann J., Isenberg D.A., Shoenfeld Y. (1986). Preferential secretion of a common anti-DNA idiotype (16/6 Id) and anti-polynucleotide antibodies by normal mononuclear cells following stimulation with Klebsiella pneumoniae. Immunol. Lett..

[190] el-Roiey A., Sela O., Isenberg D.A., Feldman R., Colaco B.C., Kennedy R.C., Shoenfeld Y. (1987). The sera of patients with Klebsiella infections contain a common anti-DNA idiotype (16/6) Id and anti-polynucleotide activity. Clin. Exp. Immunol..

[191] Riancho-Zarrabeitia L., Martínez-Taboada V., Rúa-Figueroa I., Alonso F., Galindo-Izquierdo M., Ovalles J., Olivé-Marqués A., Fernández-Nebro A., Calvo-Alén J., Menor-Almagro R., Tomero-Muriel E., Uriarte-Isacelaya E., Botenau A., Andres M., Freire-González M., Santos Soler G., Ruiz-Lucea E., Ibáñez-Barceló M., Castellví I., Galisteo C., Quevedo Vila V., Raya E., Narváez-García J., Expósito L., Hernández-Beriaín J.A., Horcada L., Aurrecoechea E., Pego-Reigosa J.M. (2020). Antiphospholipid syndrome (APS) in patients with systemic lupus erythematosus (SLE) implies a more severe disease with more damage accrual and higher mortality. Lupus.

[192] Borgnia M.J., Kozono D., Calamita G., Maloney P.C., Agre P. (1999). Functional reconstitution and characterization of AqpZ, the E. coli water channel protein. J. Mol. Biol..

[193] Ren Z., Wang Y., Duan T., Patel J., Liggett T., Loda E., Brahma S., Goswami R., Grouse C., Byrne R., Stefoski D., Javed A., Miller S.D., Balabanov R. (2012). Cross-immunoreactivity between bacterial aquaporin-Z and human aquaporin-4: potential relevance to neuromyelitis optica. J. Immunol..

[194] Gross A.J., Hochberg D., Rand W.M., Thorley-Lawson D.A. (2005). EBV and systemic lupus erythematosus: a new perspective. J. Immunol. Baltim. Md.

[195] Kang I., Quan T., Nolasco H., Park S.-H., Hong M.S., Crouch J., Pamer E.G., Howe J.G., Craft J. (2004). Defective control of latent Epstein-Barr virus infection in systemic lupus erythematosus. J. Immunol. Baltim. Md.

[196] Berkun Y., Zandman-Goddard G., Barzilai O., Boaz M., Sherer Y., Larida B., Blank M., Anaya J.-M., Shoenfeld Y. (2009). Infectious antibodies in systemic lupus erythematosus patients. Lupus.

[197] Arbuckle M.R., Reichlin M., Harley J.B., James J.A. (1999). Shared early autoantibody recognition events in the development of anti-Sm B/B’ in human lupus. Scand. J. Immunol..

[198] Poole B.D., Gross T., Maier S., Harley J.B., James J.A. (2008). Lupus-like autoantibody development in rabbits and mice after immunization with EBNA-1 fragments. J. Autoimmun..

[199] Ja J., T G., Rh S., Jb H. (1995). Immunoglobulin epitope spreading and autoimmune disease after peptide immunization: Sm B/B’-derived PPPGMRPP and PPPGIRGP induce spliceosome autoimmunity. J. Exp. Med..

[200] Calvin D.J.D., Steve R.J., Kannangai R., Abraham P., Udhaya Kumar S., Balasundaram A., George Priya Doss C., Thomas V., Thomas A., Danda D., Fletcher J.G. (2023). HPV and molecular mimicry in systemic lupus erythematosus and an impact of compiling B-cell epitopes and MHC-class II binding profiles with in silico evidence. J. Biomol. Struct. Dyn..

[201] Sève P., Ferry T., Koenig M., Cathebras P., Rousset H., Broussolle C. (2005). Lupus-like presentation of parvovirus B19 infection. Semin. Arthritis Rheum..

[202] Scofield R.H., Harley J.B. (1991). Autoantigenicity of Ro/SSA antigen is related to a nucleocapsid protein of vesicular stomatitis virus. Proc. Natl. Acad. Sci. U. S. A..

[203] Kowal C., Weinstein A., Diamond B. (1999). Molecular mimicry between bacterial and self antigen in a patient with systemic lupus erythematosus. Eur. J. Immunol..

[204] Bhardwaj T., Haque S., Somvanshi P. (2018). In silico identification of molecular mimics involved in the pathogenesis of Clostridium botulinum ATCC 3502 strain. Microb. Pathog..

[205] Lakhal S., Benabid M., Sghaier I.B., Bouratbine A., Galai Y. (2015). The sera from adult patients with suggestive signs of autoimmune diseases present antinuclear autoantibodies that cross-react with Leishmania infantum conserved proteins: crude Leishmania histone and Soluble Leishmnia antigens. Immunol. Res..

[206] Perl A., Colombo E., Dai H., Agarwal R., Mark K.A., Banki K., Poiesz B.J., Phillips P.E., Hoch S.O., Reveille J.D., Arnett F.C. (1995). Antibody reactivity to the hres-1 endogenous retroviral element identifies a subset of patients with systemic lupus erythematosus and overlap syndromes. Arthritis Rheum..

[207] Banki K., Maceda J., Hurley E., Ablonczy E., Mattson D.H., Szegedy L., Hung C., Perl A. (1992). Human T-cell lymphotropic virus (HTLV)-related endogenous sequence, HRES-1, encodes a 28-kDa protein: a possible autoantigen for HTLV-I gag-reactive autoantibodies. Proc. Natl. Acad. Sci. U. S. A..

[208] Bairkdar M., Rossides M., Westerlind H., Hesselstrand R., Arkema E.V., Holmqvist M. (2021). Incidence and prevalence of systemic sclerosis globally: a comprehensive systematic review and meta-analysis. Rheumatol. Oxf. Engl..

[209] Meyer A., Meyer N., Schaeffer M., Gottenberg J.-E., Geny B., Sibilia J. (2015). Incidence and prevalence of inflammatory myopathies: a systematic review. Rheumatol. Oxf. Engl..

[210] Pawlitzki M., Acar L., Masanneck L., Willison A., Regner-Nelke L., Nelke C., L’hoest H., Marschall U., Schmidt J., Meuth S.G., Ruck T. (2022). Myositis in Germany: epidemiological insights over 15 years from 2005 to 2019. Neurol. Res. Pract.

[211] Stolwijk C., van Onna M., Boonen A., van Tubergen A. (2016). Global prevalence of spondyloarthritis: a systematic review and meta-regression analysis. Arthritis Care Res.

[212] Brewerton D.A., Hart F.D., Nicholls A., Caffrey M., James D.C.O., Sturrock R.D. (1973). Ankylosing spondylitis and hl-a 27. Lancet.

[213] Khan M.A. (1988). Genetics of HLA-B27, Rheumatology Volume XXVII.

[214] Chatzikyriakidou A., Voulgari P.V., Drosos A.A. (2011). What is the role of HLA-B27 in spondyloarthropathies?. Autoimmun. Rev..

[215] Taurog J.D., Richardson J.A., Croft J.T., Simmons W.A., Zhou M., Fernández-Sueiro J.L., Balish E., Hammer R.E. (1994). The germfree state prevents development of gut and joint inflammatory disease in HLA-B27 transgenic rats. J. Exp. Med..

[216] Yang L., Wang L., Wang X., Xian C.J., Lu H. (2016). A possible role of intestinal microbiota in the pathogenesis of ankylosing spondylitis. Int. J. Mol. Sci..

[217] Trull A.K., Ebringer R., Panayi G.S., Colthorpe D., James D.C.O., Ebringer A. (1983). Iga antibodies to Klebsiella pneumoniae in ankylosing spondylitis. Scand. J. Rheumatol..

[218] Husby G., Tsuchiya N., Schwimmbeck P.L., Keat A., Pahle J.A., Oldstone M.B.A., Williams Jr R.C. (1989). Cross-reactive epitope with Klebsiella pneumoniae nitrogenase in articular tissue of HLA–B27+ patients with ankylosing spondylitis. Arthritis Rheum..

[219] Schwimmbeck P.L., Yu D.T., Oldstone M.B. (1987). Autoantibodies to HLA B27 in the sera of HLA B27 patients with ankylosing spondylitis and Reiter’s syndrome. Molecular mimicry with Klebsiella pneumoniae as potential mechanism of autoimmune disease. J. Exp. Med..

[220] Ahmadi K., Wilson C., Tiwana H., Ebringer A., Ahmadi K., Shanmuganathan S., Binder A., Ebringer A. (1998). Antibodies to Klebsiella pneumoniae nitrogenase reductase in patients with ankylosing spondylitis. Ann. Rheum. Dis..

[221] Fielder M., Pirt S.J., Tarpey I., Wilson C., Cunningham P., Ettelaie C., Binder A., Bansal S., Ebringer A. (1995). Molecular mimicry and ankylosing spondylitis: possible role of a novel sequence in pullulanase of *Klebsiella pneumoniae*. FEBS Lett..

[222] Cauli A., Dessole G., Vacca A., Porru G., Cappai L., Piga M., Bitti P.P., Fiorillo M.T., Sorrentino R., Carcassi C., Mathieu A. (2012). Susceptibility to ankylosing spondylitis but not disease outcome is influenced by the level of HLA-B27 expression, which shows moderate variability over time. Scand. J. Rheumatol..

[223] Rashid T., Wilson C., Ebringer A. (2013). The link between ankylosing spondylitis, Crohn’s disease, Klebsiella, and starch consumption. Clin. Dev. Immunol..

[224] Tani Y., Tiwana H., Hukuda S., Nishioka J., Fielder M., Wilson C., Bansal S., Ebringer A. (1997). Antibodies to Klebsiella, Proteus, and HLA-B27 peptides in Japanese patients with ankylosing spondylitis and rheumatoid arthritis. J. Rheumatol..

[225] Welsh J., Avakian H., Cowling P., Ebringer A., Wooley P., Panayi G., Ebringer R., spondylitis Ankylosing (1980). HLA-B27 and Klebsiella. I. Cross-reactivity studies with rabbit antisera. Br. J. Exp. Pathol..

[226] Lahesmaa R., Skurnik M., Vaara M., Leirisalo-Repo M., Nissilä M., Granfors K., Toivanen P. (1991). Molecular mimickry between HLA B27 and Yersinia, Salmonella, Shigella and Klebsiella within the same region of HLA alpha 1-helix. Clin. Exp. Immunol..

[227] Lahesmaa R., Skurnik M., Toivanen P. (1993). Molecular mimicry: any role in the pathogenesis of spondyloarthropathies?. Immunol. Res..

[228] Gumkowska-Sroka O., Kotyla K., Kotyla P. (2024). Immunogenetics of systemic sclerosis. Genes.

[229] Ko J., Noviani M., Chellamuthu V.R., Albani S., Low A.H.L. (2023). The pathogenesis of systemic sclerosis: the origin of fibrosis and interlink with vasculopathy and autoimmunity. Int. J. Mol. Sci..

[230] Kasturi K.N., Hatakeyama A., Spiera H., Bona C. (1995). Antifibrillarin autoantibodies present in systemic sclerosis and other connective tissue diseases interact with similar epitopes. J. Exp. Med..

[231] Bogdanos D.P., Sakkas L.I. (2017). From microbiome to infectome in autoimmunity. Curr. Opin. Rheumatol..

[232] Dolcino M., Puccetti A., Barbieri A., Bason C., Tinazzi E., Ottria A., Patuzzo G., Martinelli N., Lunardi C. (2015). Infections and autoimmunity: role of human cytomegalovirus in autoimmune endothelial cell damage. Lupus.

[233] Muryoi T., Kasturi K.N., Kafina M.J., Cram D.S., Harrison L.C., Sasaki T., Bona C.A. (1992). Antitopoisomerase I monoclonal autoantibodies from scleroderma patients and tight skin mouse interact with similar epitopes. J. Exp. Med..

[234] Lunardi C., Bason C., Navone R., Millo E., Damonte G., Corrocher R., Puccetti A. (2000). Systemic sclerosis immunoglobulin G autoantibodies bind the human cytomegalovirus late protein UL94 and induce apoptosis in human endothelial cells. Nat. Med..

[235] Lunardi C., Dolcino M., Peterlana D., Bason C., Navone R., Tamassia N., Beri R., Corrocher R., Puccetti A. (2006). Antibodies against human cytomegalovirus in the pathogenesis of systemic sclerosis: a gene array approach. PLoS Med..

[236] Mahler M., Mierau R., Schlumberger W., Blüthner M. (2001). A population of autoantibodies against a centromere-associated protein A major epitope motif cross-reacts with related cryptic epitopes on other nuclear autoantigens and on the Epstein-Barr nuclear antigen 1. J. Mol. Med..

[237] Kalabay L., Fekete B., Czirják L., Horváth L., Daha M.R., Veres A., Fónyad G., Horváth A., Viczián A., Singh M., Hoffer I., Füst G., Romics L., Prohászka Z. (2002). Helicobacter pylori infection in connective tissue disorders is associated with high levels of antibodies to mycobacterial hsp65 but not to human hsp60. Helicobacter.

[238] Macchia G., Massone A., Burroni D., Covacci A., Censini S., Rappuoli R. (1993). The Hsp60 protein of Helicobacter pylori: structure and immune response in patients with gastroduodenal diseases. Mol. Microbiol..

[239] Randone S.B., Guiducci S., Cerinic M.M. (2008). Systemic sclerosis and infections. Autoimmun. Rev..

[240] Carroll M., Nagarajah V., Campbell S. (2023). Systemic sclerosis following COVID-19 infection with recurrent corticosteroid-induced scleroderma renal crisis. BMJ Case Rep..

[241] Chandra A., Kahaleh B. (2022). Systemic sclerosis (SSc) after COVID-19: a case report. Cureus.

[242] Lundberg I.E., Fujimoto M., Vencovsky J., Aggarwal R., Holmqvist M., Christopher-Stine L., Mammen A.L., Miller F.W. (2021). Idiopathic inflammatory myopathies. Nat. Rev. Dis. Primer.

[243] Narayanappa G., Nandeesh B.N. (2021). Infective myositis. Brain Pathol..

[244] Megremis S., Walker T.D.J., He X., O’Sullivan J., Ollier W.E.R., Chinoy H., Pendleton N., Payton A., Hampson L., Hampson I., Lamb J.A. (2021). Analysis of human total antibody repertoires in TIF1γ autoantibody positive dermatomyositis. Commun. Biol..

[245] Guéry J.-C. (2019). Why is systemic lupus erythematosus more common in women?. Joint Bone Spine.

[246] Intriago M., Maldonado G., Cárdenas J., Ríos C. (2019). Clinical characteristics in patients with rheumatoid arthritis: differences between genders. Sci. World J..

[247] Linos A., Worthington J.W., O’Fallon W.M., Kurland L.T. (1980). The epidemiology of rheumatoid arthritis in Rochester, Minnesota: a study of incidence, prevalence, and mortality. Am. J. Epidemiol..

[248] Hughes M., Pauling J.D., Armstrong-James L., Denton C.P., Galdas P., Flurey C. (2020). Gender-related differences in systemic sclerosis. Autoimmun Rev..

[249] Bjornevik K., Cortese M., Healy B.C., Kuhle J., Mina M.J., Leng Y., Elledge S.J., Niebuhr D.W., Scher A.I., Munger K.L., Ascherio A. (2022). Longitudinal analysis reveals high prevalence of Epstein-Barr virus associated with multiple sclerosis. Science.

[250] Cortese M., Leng Y., Bjornevik K., Mitchell M., Healy B.C., Mina M.J., Mancuso J.D., Niebuhr D.W., Munger K.L., Elledge S.J., Ascherio A. (2024). Serologic response to the epstein-barr virus peptidome and the risk for multiple sclerosis. JAMA Neurol..

[251] Yuan F., Wei F., Huang H., Xue Y., Guo P., You Y. (2018). The predictive value of autoantibody spectrum on organ damage in patients with systemic lupus erythematosus. Arch. Rheumatol..

[252] Maple P.A., Ascherio A., Cohen J.I., Cutter G., Giovannoni G., Shannon-Lowe C., Tanasescu R., Gran B. (2022). The potential for EBV vaccines to prevent multiple sclerosis. Front. Neurol..

[253] Scarpini S., Morigi F., Betti L., Dondi A., Biagi C., Lanari M. (2021). Development of a vaccine against human cytomegalovirus: advances, barriers, and implications for the clinical practice. Vaccines.

[254] O’Brien-Simpson N.M., Holden J.A., Lenzo J.C., Tan Y., Brammar G.C., Walsh K.A., Singleton W., Orth R.K.H., Slakeski N., Cross K.J., Darby I.B., Becher D., Rowe T., Morelli A.B., Hammet A., Nash A., Brown A., Ma B., Vingadassalom D., McCluskey J., Kleanthous H., Reynolds E.C. (2016). A therapeutic Porphyromonas gingivalis gingipain vaccine induces neutralising IgG1 antibodies that protect against experimental periodontitis. NPJ Vaccines.

[255] Balakrishnan B., Taneja V. (2018). Microbial modulation of the gut microbiome for treating autoimmune diseases. Expert Rev. Gastroenterol. Hepatol..

[256] Sawalha A.H., Schmid W.R., Binder S.R., Bacino D.K., Harley J.B. (2004). Association between systemic lupus erythematosus and Helicobacter pylori seronegativity - PubMed. J. Rheumatol..

[257] Chen M., Aosai F., Norose K., Mun H.-S., Ishikura H., Hirose S., Piao L.-X., Fang H., Yano A. (2004). Toxoplasma gondii infection inhibits the development of lupus-like syndrome in autoimmune (New Zealand Black x New Zealand White) F1 mice. Int. Immunol..

[258] He Z., Kong X., Shao T., Zhang Y., Wen C. (2019). Alterations of the gut microbiota associated with promoting efficacy of prednisone by bromofuranone in MRL/lpr mice. Front. Microbiol..

[259] Liu X., Liu M., Zhao M., Li P., Gao C., Fan X., cai G., Lu Q., Chen X. (2023). Fecal microbiota transplantation for the management of autoimmune diseases: potential mechanisms and challenges. J. Autoimmun..

[260] Yang R., Chen Z., Cai J. (2023). Fecal microbiota transplantation: emerging applications in autoimmune diseases. J. Autoimmun..

[261] Al-Fakhrany O.M., Elekhnawy E. (2024). Next-generation probiotics: the upcoming biotherapeutics. Mol. Biol. Rep..

[262] Murali S.K., Mansell T.J. (2024). Next generation probiotics: engineering live biotherapeutics. Biotechnol. Adv..

[263] Suez J., Zmora N., Segal E., Elinav E. (2019). The pros, cons, and many unknowns of probiotics. Nat. Med..

[264] Patangia D.V., Anthony Ryan C., Dempsey E., Paul Ross R., Stanton C. (2022). Impact of antibiotics on the human microbiome and consequences for host health. MicrobiologyOpen.

[265] Vieira S.M., Hiltensperger M., Kumar V., Zegarra-Ruiz D., Dehner C., Khan N., Costa F.R.C., Tiniakou E., Greiling T., Ruff W., Barbieri A., Kriegel C., Mehta S.S., Knight J.R., Jain D., Goodman A.L., Kriegel M.A. (2018). Translocation of a gut pathobiont drives autoimmunity in mice and humans. Science.

[266] Crost E.H., Coletto E., Bell A., Juge N. (2023). Ruminococcus gnavus: friend or foe for human health. FEMS Microbiol. Rev..

[267] Hajishengallis G., Lamont R.J., Koo H. (2023). Oral polymicrobial communities: assembly, function and impact on diseases. Cell Host Microbe.

[268] Stölzel U., Schuppan D., Tillmann H.L., Manns M.P., Tannapfel A., Doss M.O., Zimmer T., Köstler E. (2002). Autoimmunity and HCV infection in porphyria cutanea tarda: a controlled study. Cell. Mol. Biol. Noisy--Gd. Fr.

[269] Ribeiro F.M., Gomez V.E., Albuquerque E.M.N., Klumb E.M., Shoenfeld Y. (2015). Lupus and leprosy: beyond the coincidence. Immunol. Res..

[270] Smatti M.K., Cyprian F.S., Nasrallah G.K., Al Thani A.A., Almishal R.O., Yassine H.M. (2019). Viruses and autoimmunity: a review on the potential interaction and molecular mechanisms. Viruses.

[271] Ramos-Casals M., Loustaud-Ratti V., De Vita S., Zeher M., Bosch J.-A., Toussirot E., Medina F., Rosas J., Anaya J.-M., Font J. (2005). the S.-H.S. Group, Sjögren syndrome associated with hepatitis C virus: a multicenter analysis of 137 cases. Medicine..

[272] Pacheco Y., Acosta-Ampudia Y., Monsalve D.M., Chang C., Gershwin M.E., Anaya J.-M. (2019). Bystander activation and autoimmunity. J. Autoimmun..

