# Supplementary material for: Molecular mimicry in the pathogenesis of autoimmune rheumatic diseases
Source: J Transl Autoimmun. 2025 Jan 7;10:100269. doi: 10.1016/j.jtauto.2025.100269 (PMC11773492; doi:10.1016/j.jtauto.2025.100269)
Supplement: Multimedia component 1 [file mmc1.docx]

|  | **RA** | | | **SLE** | | | **AS** | | | **SSc** | | | **Myositis** | | |
| --- | --- | --- | --- | --- | --- | --- | --- | --- | --- | --- | --- | --- | --- | --- | --- |
|  | *Prev* | *Details* | *Ref* | *Prev* | *Details* | *Ref* | *Prev* | *Details* | *Ref* | *Prev* | *Details* | *Ref* | *Prev* | *Details* | *Ref* |
|  | 0.46% | Global | [1] | 0.10% | Global | [2] | 0.18% | global | [3] | 0.02% | Global | [4] | 0.06% | Germany | [5] |
|  | 0.80% | Global (validated by rheumatologists) | [1] | 0.44% | Global | [6] |  |  |  | 0.02% | Global | [7] | 0.02% | Global, for inflammatory myopathies | [8] |
| **Ø** | **0.63%** |  |  | **0.27%** |  |  | **0.18%** |  |  | **0.02%** |  |  | **0.04%** |  |  |

***
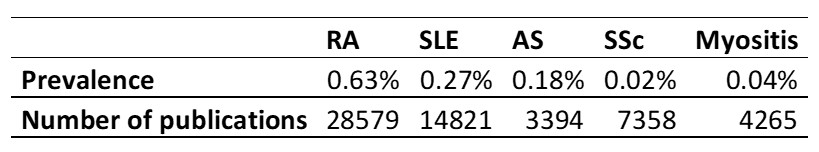
****
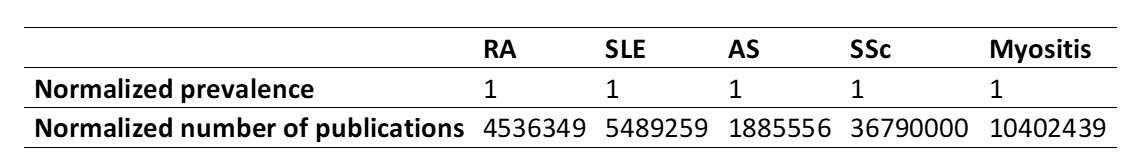
****
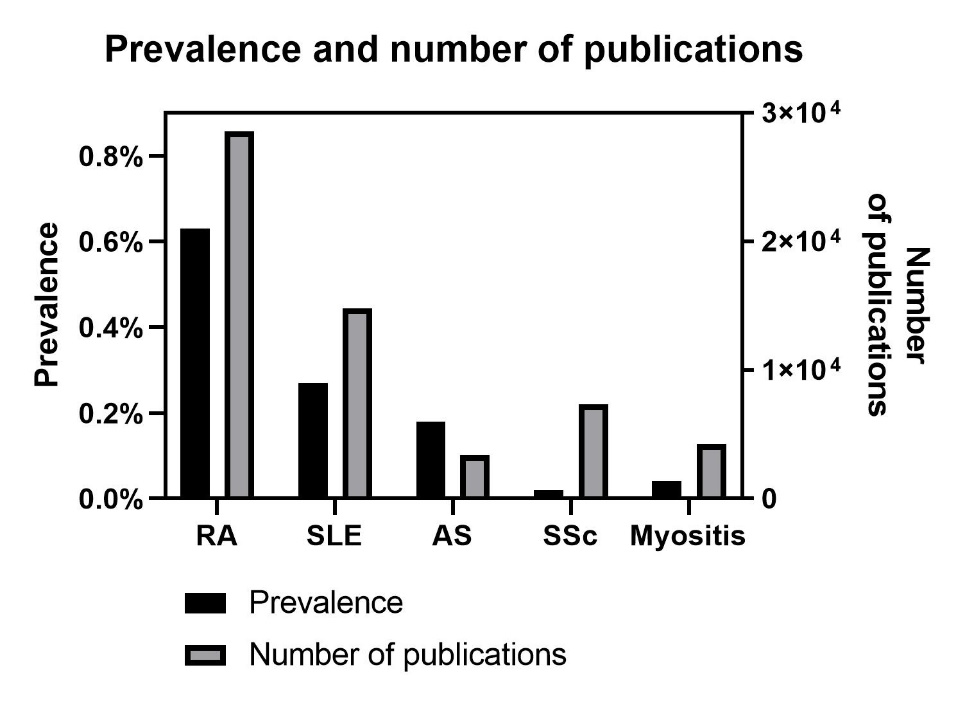
***
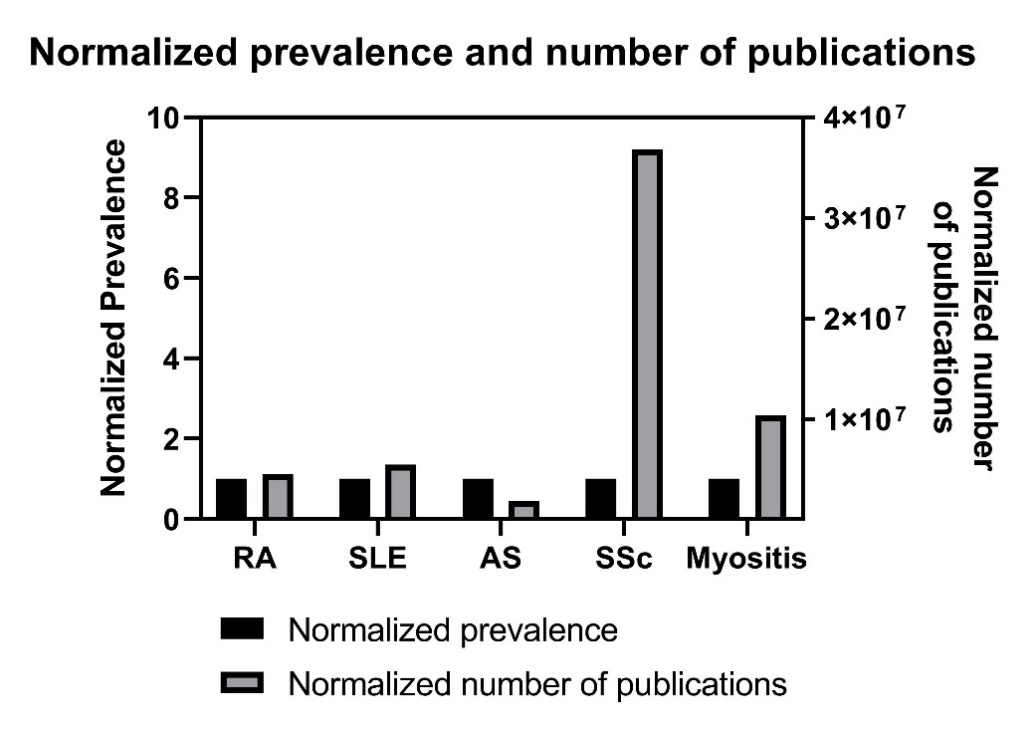


**A**

**B**

***Supplementary Figure 1: Comparison of disease prevalence and number of publications for different autoimmune rheumatic diseases. A.*** *Prevalence values (Prev) for rheumatoid arthritis (RA), systemic lupus erythematosus (SLE), ankylosing spondylitis (AS), systemic sclerosis (SSc) and myositis were obtained from the references (Ref) as indicated, and average values (Ø) were calculated. Details describe the study population and any other relevant information.* ***B.*** *To assess research output, a PubMed search was conducted on October 18, 2024, using full disease names: RA, SLE, AS, SSC and myositis. For normalized comparison, prevalence was set to 1 and number of publications adjusted by the normalization factor.*

**Supplementary references**

[1] K. Almutairi, J. Nossent, D. Preen, H. Keen, C. Inderjeeth, The global prevalence of rheumatoid arthritis: a meta-analysis based on a systematic review, Rheumatol Int 41 (2021) 863–877. https://doi.org/10.1007/s00296-020-04731-0.

[2] F. Fatoye, T. Gebrye, C. Mbada, Global and regional prevalence and incidence of systemic lupus erythematosus in low-and-middle income countries: a systematic review and meta-analysis, Rheumatol Int 42 (2022) 2097–2107. https://doi.org/10.1007/s00296-022-05183-4.

[3] C. Stolwijk, M. van Onna, A. Boonen, A. van Tubergen, Global Prevalence of Spondyloarthritis: A Systematic Review and Meta-Regression Analysis, Arthritis Care Res (Hoboken) 68 (2016) 1320–1331. https://doi.org/10.1002/acr.22831.

[4] M. Bairkdar, M. Rossides, H. Westerlind, R. Hesselstrand, E.V. Arkema, M. Holmqvist, Incidence and prevalence of systemic sclerosis globally: a comprehensive systematic review and meta-analysis, Rheumatology (Oxford) 60 (2021) 3121–3133. https://doi.org/10.1093/rheumatology/keab190.

[5] M. Pawlitzki, L. Acar, L. Masanneck, A. Willison, L. Regner-Nelke, C. Nelke, H. L’hoest, U. Marschall, J. Schmidt, S.G. Meuth, T. Ruck, Myositis in Germany: epidemiological insights over 15 years from 2005 to 2019, Neurol Res Pract 4 (2022) 62. https://doi.org/10.1186/s42466-022-00226-4.

[6] J. Tian, D. Zhang, X. Yao, Y. Huang, Q. Lu, Global epidemiology of systemic lupus erythematosus: a comprehensive systematic analysis and modelling study, Annals of the Rheumatic Diseases 82 (2022) 351. https://doi.org/10.1136/ard-2022-223035.

[7] J. Tian, S. Kang, D. Zhang, Y. Huang, M. Zhao, X. Gui, X. Yao, Q. Lu, Global, regional, and national incidence and prevalence of systemic sclerosis, Clin Immunol 248 (2023) 109267. https://doi.org/10.1016/j.clim.2023.109267.

[8] A. Meyer, N. Meyer, M. Schaeffer, J.-E. Gottenberg, B. Geny, J. Sibilia, Incidence and prevalence of inflammatory myopathies: a systematic review, Rheumatology (Oxford) 54 (2015) 50–63. https://doi.org/10.1093/rheumatology/keu289.
